# Early visual language skills affect the trajectory of literacy gains over a three-year period of time for preschool aged deaf children who experience signing in the home

**DOI:** 10.1371/journal.pone.0229591

**Published:** 2020-02-27

**Authors:** Thomas E. Allen, Donna A. Morere

**Affiliations:** 1 Science of Learning Center on Visual Language and Visual Learning, Gallaudet University, Washington, DC, United States of America; 2 PhD in Educational Neuroscience Program, Gallaudet University, Washington, DC, United States of America; 3 Department of Psychology, Gallaudet University, Washington, DC, United States of America; University of Massachusetts - Dartmouth, UNITED STATES

## Abstract

Previous research has established a correlation between literacy skills and sign language skills among deaf children raised in signing families, but little research has examined the impact of early signing skills on the *rate of growth* of emergent literacy in early childhood. A subset of data was extracted from a larger dataset containing national longitudinal data from a three-year investigation of early literacy development of deaf children who were between the ages of three and six at the outset of the study. Selection criteria for inclusion in this limited sample included: 1) being rated as having little or no access to spoken language and 2) being raised in homes in which signs were regularly used as a means of communication (N = 56). Our purpose was twofold: 1) to examine and describe the trajectories of growth in letter and word identification skill for this sample in relation to the participants’ initial ages; and 2) to assess the degree to which the presence or deaf parents in the home (DoD) and the receptive American Sign Language (ASL) skills of the participants impacted both the level of emerging print literacy and its rate of growth over the three year period. We hypothesized that both the presence of a deaf parent in the home and the acquisition of ASL skills, a strong native language, would contribute to both the overall letter and word identification skills and to the rates of growth of this skill over time. Results indicated that having a deaf parent did, indeed, impact emergent literacy attainment, but its effect was rendered nonsignificant when ASL skill was taken into consideration. Possession of stronger ASL skills, whether or not the children had deaf parents, contributed significantly to both the levels and rate of growth. The findings contribute to the body of work that emphasizes the importance early language skills (spoken or signed) to later academic success and dispels the myth that deaf children with deaf parents have exclusive access to the acquisition of these skills.

## Introduction

A vast body of research has documented the importance of language to the development of literacy. Much of this research has focused on the relationship between the emergence of skills in spoken language in the preschool years and subsequent gains in the achievement of reading skills. [1, 2] Given the degree to which the individual sounds of spoken words can be mapped onto the letters of the alphabet (and therefore onto the printed words on a page), it has often been claimed that mastery of a spoken language is a prerequisite to the mastery of reading. Yet this claim does not explain the development of reading skill among children who are deaf and who acquire mastery of a visual language with little or no access to the sounds of spoken language. Deaf children represent a diverse group of learners for whom print literacy development has proven challenging, with consistently low outcomes noted over decades of testing in the United States [3], a finding that has been replicated in deaf populations across languages [4, 5]. While some deaf children do develop strong literacy skills, the majority develop limited print literacy [3]. The question this raises is how do deaf children develop strong print literacy skills and how can this development be supported.

In recent years, some researchers have turned their attention to the literacy development of deaf children and the apparent contradiction to the commonly held beliefs regarding the inseparability between the mastery of a sound-based language and the development of reading skills. For example, studies of infant brain development [e.g., 6] have noted that the regions of the brain active in the processing of sound-based phonology in hearing infants are precisely those that are active in the processing of a visually based phonology in the acquisition of a sign language, such as American Sign Language (ASL). This research suggests the possibility that it is the development these language regions during infancy that is critical to the emergence of phonological knowledge (spoken or visual), which, in turn, is a prerequisite to the development of reading skills, independent of the modality of language input. [7] Additionally, a growing number of behavioral studies have documented strong associations between ASL skills and reading skills in samples of deaf participants [e.g., 8, 9].

The literature cited above has limitations. First, neuro-imaging studies were conducted in laboratories with controlled conditions and small, relatively homogeneous samples, limiting generalizability. Second, correlational studies were not able to probe causal relationships among language exposure, language skill, and reading skill, and these studies employed cross-sectional designs that were not able to identify developmental patterns and growth trajectories of deaf children from signing families as they progress through their preschool years and into elementary schools. Third, little attention in these studies was given to articulating individual differences and evaluating subgroups. Longitudinal studies involving signing deaf children are needed to better understand the trajectories of academic growth and the factors, such as parental hearing status and use of a visual language, that impact those trajectories.

The majority of existing longitudinal studies have addressed early print literacy development of deaf children, such as those by the Colin, Easterbrooks, and Harris and their colleagues, generally have small samples [10–14], and they vary considerably in terms of the participants’ level of hearing loss, itself a strong correlate of reading skill [15]. This confound frequently affects studies that include deaf children whose primary communication is spoken language, as these children often have greater levels of residual hearing than those who depend on signs and greater residual hearing is often associated with more age-appropriate reading skills [13]. (Within this paper the terms “signs” and “signing” are used as generic references to all types of signing, including natural signed languages, artificially constructed sign systems designed to represent spoken languages, and pidgin, or contact, signs. The term “sign language” is reserved for natural signed languages.) Even so, language skills appear to best predict reading outcomes. For example, Worsfold and colleagues studied older British children with hearing losses ranging from moderate (40–69 dB) to profound (≥95 dB) who used spoken language (n = 53) and found that spoken language skills at age eight best predicted reading comprehension in adolescence [16]. While the relationship between spoken language skills and reading outcomes is not surprising, it does support the contention that underlying language skills are strongly associated with print literacy development in deaf, as well as hearing, children. Another recent study of British children (n = 41) by Harris and colleagues had both oral (44%) and signing (37%) children (the remainder used mixed speech and signs) beginning at age six, but neither British Sign Language (BSL) skills nor the effects of degree of hearing loss were examined [17]. The higher performing children also generally had their hearing loss diagnosed at an earlier age, and they more often used speech as their primary means of communication. Thus, the inclusion of both deaf and hard-of-hearing children in a single small sample and the reporting of advantages accruing to participants with stronger spoken language skills limits the generalizability of findings to children with little usable hearing and an inability to hear and understand words. These factors limit the generalizability of each study’s findings as they relate to early language impacts on reading, particularly for children whose primary means of communication involves signs with or without spoken language support.

While studies such as these support the contention that better spoken language skills are associated with better print literacy outcomes, they do not address the effects of sign language skills on print literacy. Such clarification will come only through longitudinal research designs that are conducted with children with limited access to spoken language who are exposed to a visual language early in life.

The current study remedies these limitations through employing an extant longitudinal data base, from which data from a specific subgroup were extracted, i.e., deaf children between the ages of three and six with significantly limited functional hearing ability and being raised in homes in which signs were regularly used as a means of communication. The children were followed over a three-year period, during which their signing skills and emergent literacy skills were assessed.

### The role of early sign language development and emergent literacy

For hearing children, a considerable body of recent research has demonstrated the importance of early spoken language on later literacy [18–20]. However, studies of early visual language and its impact on literacy for deaf children are far less prevalent. Instead, studies of the impact of sound-based phonological coding and awareness and phonological knowledge on reading are more common in the deaf education literature [21]. A more limited number of studies, such as those by Harris and colleagues and Mayberry and Eichen, have considered or explored alternative factors, such as a deaf child’s exposure to and mastery of a visual language or speechreading skills [17, 22]. Studies of older American deaf children and deaf adults have repeatedly noted strong correlational evidence for a relationship between ASL skill and reading skill [8, 9, 23–26]. This relationship has not been studied adequately among deaf children in their pre-school years.

Lederberg and colleagues studied a large (n = 336) sample of American (from nine US states and one Canadian province) deaf and hard of hearing schoolchildren in kindergarten (age 5) through second grade [27]. This study was investigating underlying factors associated with early reading skills and divided the children into three groups based on communication method. One group (Spoken, n = 101) was identified as having auditory access to language and having only spoken language used by both parents and teachers. A second group (Unimodal Sign, n = 131) was identified as having little to no speech perception regardless of amplification and teachers who used signs with or without speech. The third group (Bimodal, n = 104) was identified as having auditory access to spoken language and teachers who sign with or without speech. One hundred nineteen of the participants had CIs; 57 of the remainder had unaided hearing losses in the mild to moderate range, 72 had moderately severe to severe, and 67 had profound hearing loss. Although the degrees of hearing loss were not broken down across the three groups, demographic data reported that all but one of the children in the Spoken Language only group used CIs and/or hearing aids and all but one, who had some word identification, had consistent auditory word identification. Furthermore, the majority of this group (53%) had no reported speech articulation impairment and only 2% were reported to have severe impairment. In contrast, the Unimodal Sign group was reported to have negligible speech skills and no auditory word identification skills. The Bimodal group was reported to have speech perception skills nearly comparable to those of the Spoken Language group, but more variable speech skills, with a quarter having no impairment and a third having severe impairment. Thus, these groups have varying degrees of access to spoken and signed communication. The form of sign communication varied, with 62% of the teachers reporting using ASL only, 27% reporting using both ASL and Signed English, and 11% reporting using only Signed English, apparently across both groups of signing children.

Lederberg and colleagues administered a set of measures to the children individually during a single school term [27]. The Unimodal Sign group was not administered tasks requiring spoken responses (e.g., sound blending), while sign-based tasks (e.g., Fingerspelling Blend) were not administered to the Spoken Language only group. The analyses indicated similar patterns across the three groups, but differences in how these patterns occurred. There was a significant underlying relationship between reading and both underlying language (signed or spoken) and the ability to manipulate components of words for all three groups. The relationships between spoken language phonology and both language and reading was similar for the Spoken Language group and Bimodal groups, but the direct influence of language on print literacy appeared stronger on the latter. For the Unimodal Sign group, the strongest relationship was between language (ASL) and print literacy. Fingerspelling analysis skills (e.g., Blend and Elision) also had a strong relationship with print literacy for the two signing groups, loaded into the Literacy factor. This suggests that while deaf children with access to spoken language may depend heavily on spoken language phonology for print literacy development, underlying language skills (e.g., ASL) may be more crucial for deaf and hard of hearing children who depend on signed communication.

A number of studies from Mayberry and colleagues and others have supported the contention that early language access via ASL supports literacy development [21, 28, 29]. Furthermore, research by Holmer and colleagues and McQuarrie and Abbott has suggested that better sign-based phonological awareness may be associated with higher levels of reading skills [30, 31]. As with other aspects of early literacy development, elucidation of the relationships between early ASL skills and early literacy development is needed.

Scott and Hoffmeister studied deaf and hard of hearing secondary students in bilingual ASL and English programs in the United States [32]. While they found that academic English knowledge and word reading fluency predicted reading comprehension outcomes, consistent with the above outcomes, ASL proficiency was the strongest predictor of reading comprehension performance. Furthermore, Novogrodsky and colleagues found that in deaf students ages four to 18, knowledge of ASL antonyms eliminated the advantage of having deaf parents on reading comprehension outcomes, again suggesting that it is the underlying ASL skills rather than parental hearing status that drives the enhanced reading outcomes [33]. Novogrodsky’s group also found that deaf children of hearing parents had similar trajectories of ASL development to deaf children of deaf parents, but the skill development was delayed [34]. Based on the outcomes indicating that ASL skills support reading skill development, Andrews and colleagues and Hoffmeister and Caldwell-Harris developed models for alternative pathways to reading skill development based on prior knowledge of ASL for deaf signers [35, 36].

In a cross-sectional analysis of children from the first year of data collection of this project, Allen [37] examined the impact of early visual language experience on the letter-writing skills of deaf children ages three to five. This analysis demonstrated significant effects of both ASL receptive skills and fingerspelling skills (controlling for age) on a child's ability to write the letters of the alphabet [37]. In a linear regression path model, ASL skill had both a direct effect on letter writing skill, controlling for age and fingerspelling skills, and an indirect effect through its impact on fingerspelling skill. The three-variable model explained 58% of the variance in letter writing. The results indicated that early ASL exposure, leading to increased language skills, and home or school-based training in fingerspelling are potent predictors of early print-based literacy among deaf children. Furthermore, the results suggest that ASL contributes to important emergent literacy skills, independent of fingerspelling. The above studies, which examined natural sign languages as first languages, support the contention that, at least in analyses of concurrent testing, a strong first language in a natural sign language supports print literacy development. The current study extends this work by examining the impacts of these early visual language factors on the trajectories of emergent print literacy acquisition in pre-school aged deaf children using a longitudinal design.

### Trajectories of deaf children’s reading skills

While the numbers of participants in previous longitudinal studies of early literacy development in deaf children have been small, limiting the depth of analysis possible, they have produced some interesting findings. One finding is the varied trajectories of literacy development in these deaf samples, many of which differ from those seen in the general population. Studies of British children by Harris and colleagues [13, 14, 17] have found that while the initial literacy development of the deaf children resembled that of the hearing children, the progress of the deaf children appeared to slow over time relative to their hearing peers. Consistent with previous research [38], the trajectories of the literacy development of the deaf and hearing cohorts diverged, with the deaf children failing to transition from early to more advanced reading skills. Indeed, in some analyses, the reading comprehension of the deaf students did not increase between the second and third years of the study [17]. One issue with these studies is the wide range of residual hearing and inclusion of children whose communication is primarily spoken language. There is a critical need for studies that evaluate language skills in subgroups that are more homogeneous with respect to the level of hearing and communication. This is one reason that the current study focused on children who were reported to have limited access to spoken language and used signs in the home rather than the children whose parent survey indicated that they could hear and understand spoken language and whose communication in the home environment was limited to spoken language.

One factor to take into consideration when investigating the relationship between visual language skills and educational outcomes is what respondents mean when they report the use of “signing” or a specific signed language. Reporting a primary use of signing in the home itself does not necessarily suggest that the participants possessed advanced skills in the natural signed language of the environment (e.g., BSL, ASL, etc.), a language with unique grammar and syntax, rather than simply signed communication, which may use signs borrowed from natural signed languages, but may focus on spoken language grammar and syntax. Indeed, reporting of use of signs may even represent occasional use of individual signs to clarify speech or a pidgin-like combination of English and ASL or other spoken and signed language pairs, thus lacking full linguistic representation of either language. Parents may report using ASL or another signed language in the home although they may use more English (or other spoken language) structure whether or not they voice when they sign. This may be true even of deaf parents who were raised with more English oriented signing in their homes of origin. Thus, simply having exposure to signs (or even “ASL”) in the home does not necessarily correspond to the child developing a linguistically accurate first language based on that exposure.

This leaves open the critical possibility that children with demonstrated skills in natural signed languages such as ASL might show higher overall levels of literacy attainment and steeper trajectories of reading gain than those with lower ASL skill levels. Further research with more homogeneous samples of children is required to investigate these relationships. Thus, the current study focuses on children who are reported as being raised in families that regularly use signs at home with or without speech. Due to the previously reported changes in trajectories over time, in addition to investigating both the levels of performance and the overall trajectories of deaf children ages three to eight, this study investigates whether the age at first testing affects the print literacy trajectories.

### Parental hearing status

Deaf children whose parents are deaf are commonly reported to demonstrate more age appropriate language skills than their deaf peers with hearing parents [39]. This is attributed to the availability of early accessible language, as a range of research reviewed by Lederberg and colleagues indicates that deaf children whose deaf parents are fluent ASL signers develop ASL skills in a manner that parallels the spoken language development of hearing children [39]. Studies have demonstrated such parallels for articulation (manual articulation of signs paralleling the development of spoken language articulation), vocabulary, and levels of grammatical complexity. It has been suggested that, based on the research indicating that early language skills predict reading outcomes for deaf students, this would be expected to result in an early reading advantage for deaf children with deaf parents. However, those researchers noted that while more advanced sign skills were reported, the finding of more advanced reading in skills in deaf children with deaf parents has not been a consistent outcome. Indeed, DeLana and colleagues reported a significant relationship between ASL skills and reading achievement in a group of American deaf students regardless of hearing status of their parents and did not find a significant effect based on having deaf parents or siblings [40]. Similarly, Novogrodsky and colleagues found that it was the deaf students’ knowledge of ASL antonyms rather than parental hearing status that affected reading comprehension outcomes [33]. Thus, it appears likely that good first language skills may be necessary for appropriate reading skill development, but simply having a deaf parent may not be predictive of more advanced reading skills. Thus, the effect of parental hearing status on longitudinal reading outcomes was investigated in the current study.

### Research questions

1. **What are the trajectories of growth in letter and word identification skills over three years among signing deaf children who were between the ages of 3 and 6 in Wave 1?**
  We hypothesize that deaf children gain skills over time, but that the average rates of gain may differ for children of different ages and the normative comparisons of deaf children with hearing children may show different trajectories as children enter elementary school.
2. **To what extent do the presence of a deaf parent in the home and the level of American Sign Language skill (uniquely and in combination) contribute to the overall level and to the rate of growth in letter word identification?**
  We hypothesize that the presence of a deaf parent in the home and the level of ASL Skill will both contribute to emerging letter word identification skills, but that the level of ASL Skills, indicating the acquisition of a native language, will be the stronger predictor.

## Materials and methods

In order to study the trajectories of growth in emergent literacy among young deaf children who were making the transition from pre-school to elementary school (and the student, home, and school factors that contribute to these trajectories), the current paper presents an analysis of a subset of data from the Early Education Longitudinal Study (EELS), a three-year study designed to provide a large database of a national sample which could be accessed by both the study designers and other researchers to investigate a range of aspects of the development of young deaf children conducted within the National Science Foundation Science of Learning Center on Visual Language and Visual Learning (VL2) from 2009 to 2012. A full description of the EELS instrumentation, procedures, and sample characteristics is available in in a technical report [41].

Data from both direct assessments (administered at the child’s school, with all but the ASL skills assessment administered using the child’s communication preference) and the survey completed by the parent who enrolled their child in the study were used for the current study. The following inclusion criteria were used for the study1) between the ages of three and five at the beginning of the 2010–2011 school year; 2) hearing thresholds in the severe to profound range; and 3) no learning or cognitive impairments that would prohibit their participation in the study. The Gallaudet Institutional Review Board reviewed and approved the research, including all forms and surveys, prior to recruitment of participants. The parent or guardian who registered the child for participation in the study was asked to complete the informed consent form as part of the registration process. Not all parents who provided informed consent completed the survey; thus, some children who were evaluated did not have the associated demographic data and thus could not be included in the current analyses. Children without completed informed consent forms on file were not included in the study; however, those with consent but without parent surveys were included in the total number of participants in the full EELS study.

### Characteristics of study sample

The focus of the current study was on the potential effects of early visual language experience on print literacy development. Thus, the subset of participants for the current study (targets), i.e., children from ages three to five with limited functional hearing for whom it was reported that signing was regularly used in the family, were selected from the larger EELS dataset (total sample).

#### Signing deaf children

As most early language exposure occurs in the home, it was decided to use parent reports of signed communication being used regularly in the home for inclusion in the target sample. As it was reported that signing was regularly used in the home, these families will be considered signing families regardless of the form of singing reported, whether spoken language was also used, or the type of communication used at school. A wide range of forms of signing are used by families in the US, including ASL and a various English-based sign systems, with or without accompanying speech the (former of which may be referred to as sign supported speech). Even families who attempt and report using ASL may use more English-orient signing some or most of the time. Without direct observation of the signing in the home, it is impossible to determine the exact nature of the signing that is actually used. Thus, the decision was made to include all children whose parents reported regular use of singing in the home, regardless of the form of the signing, in these analyses. These children will be referred to as signing deaf children and reflect the types of children in schools and families in the US who report using signing in the home.

#### Functional hearing level

It was not possible to obtain audiological data from study participants. Instead, the EELS parent questionnaire included an adaptation of the Gallaudet Hearing Scale [42]. As seen below in the table of deaf-related demographic characteristics, this scale consists of a number of statements regarding a respondent’s functional ability to hear and understand speech under a variety of conditions ranging from “…*when whispered to across a quiet room*” to “…*when spoken loudly in my child’s better ear*”. For respondents who cannot hear and understand speech even in the “spoken loudly” condition, three additional statements are provided: “…*can usually tell one kind of noise from another”*, *“*..*can hear loud noises”*, and *“*..*cannot hear anything at all*.” In the EELS study, parents responded on behalf of their child, e.g., “*My child cannot hear anything at all*.” The Gallaudet Scale presents the statements in decreasing levels of functional hearing ability, and an ordinal level scale value is assigned indicating the participant’s level of functional hearing. As noted above, the current study explores the role of signing in facilitating the acquisition of emerging literacy skills among children with functional hearing limited enough to provide little or no access to spoken language. As such, the Gallaudet Scale was used to help ensure that our target sample contained only those participants from the larger EELS study met the selection criteria regarding their functional unaided access to spoken language.

Due to the scheduling of testing, a small number of participants were six years of age at the time of their initial testing. In the analyses to follow, these participants are listed as a separate cohort (C4). Participants from the EELS study from oral families, and those identified as hard of hearing (scoring from 1 to 3 on the Gallaudet Scale) were intentionally excluded, ensuring a more homogeneous sample commensurate with the theoretical framework driving the analysis.

When we were designing the EELS study, we underestimated the challenges of collecting assessment data and family data for a pre-school aged population that was exceedingly diverse, geographically dispersed, and of low prevalence. Additionally, tracking preschoolers for three years, administering a battery of assessments in each year, and asking parents to complete study questionnaires in each of the three years as well, resulted in a data base with missing data and mismatched assessment and survey records. This was a national sample; thus, even when parental consent to evaluate the children was provided, children were not always available for testing when the assessment team was in town to perform the assessments. Additionally, some children who were evaluated were unable to complete the testing due to time constraints, technical difficulties (e.g., a broken test DVD), or other reasons.

To maximize the use of available data in creating a target sample for the planned analyses, we imputed missing Letter Word Identification (LWI) scores for participants who were missing only one of the three waves of LWI scores and who had bone fide LWI assessments in Wave 3. Given the high intercorrelations among the three waves of LWI assessment data, we regressed Wave 1 scores on Waves 2 and 3, substituting a predicted score for those missing Wave 1 assessments, and we regressed Wave 2 scores on Wave 1 and Wave 3, substituting a predicted score for those missing Wave 2 assessments.

To maximize the parental background information, we examined the Wave 2 and Wave 3 background surveys, transferring data elements from the Wave 2 and Wave 3 files when these elements were missing from the Wave 1 parent survey dataset. We note that not all elements were asked in all three years. For example, race and ethnicity were only asked in Wave 1.

**Fig 1:**
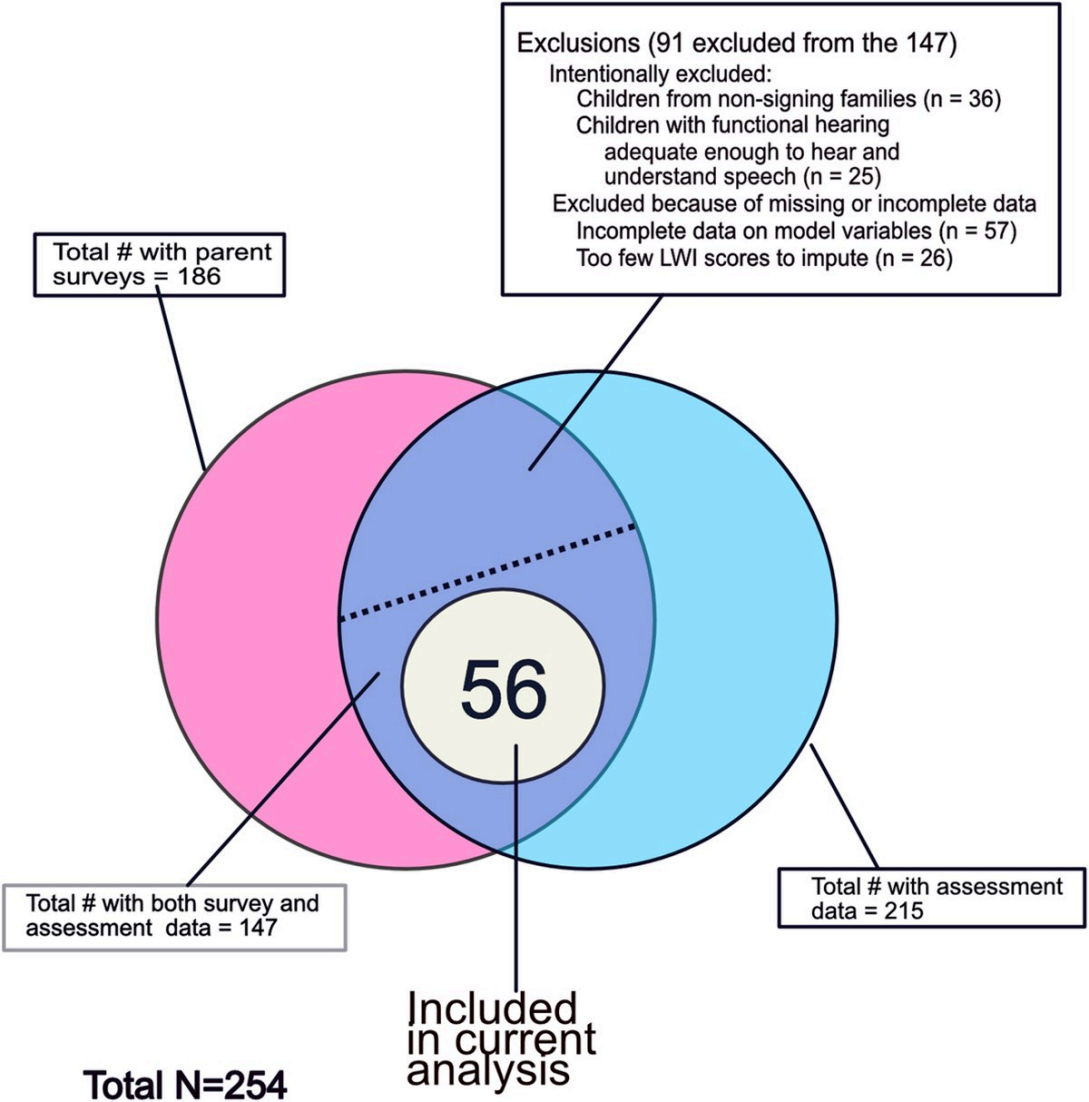
Venn diagram indicating the relationship of the target sample to the full sample.

The different components of the EELS database are presented as a Venn Diagram in Fig 1. As can be seen in this figure, the number of participants with both assessment and family background data was 147 (out of a total participant pool of 254 with data from any source). After excluding participants who: 1) were non-signers and/or were reported to have functional hearing levels sufficient to hear and understand speech without amplification; 2) had too few LWI assessments for imputation; and 3) had incomplete information on model variables, namely, parental hearing status and ASL Receptive Skills test scores, the resulting target sample contained 56 participants. Note that the total number of exclusions listed in Fig 1 under the intentional or missing information categories sum to greater than 91 because some participants were excluded under multiple categories. The analyses presented below will be limited to these 56 participants. What follows is a brief analysis of the characteristics of this target sample.

Table 1 shows the distributions of selected demographic characteristics for both the full EELS sample of 186 participants with family background surveys and the 56 participants that comprise the target sample. With the exception of race/ethnicity—69.3% of the participants with background data in the “White, not of Hispanic origin” category compared to 83.0% in the target sample–the target and full samples are highly comparable in the proportional distributions of Age, Sex, and Income.

**Table 1:** Comparisons of full EELS and target sample characteristics (demographic).

		All survey participants		Target sample	
		N	%	N	%
Age at Wave 1	Three	53	29.9%	15	26.8%
	Four	54	30.5%	16	28.6%
	Five	58	32.8%	19	33.9%
	Six	12	6.8%	6	10.7%
	Total	177	100.0%	56	100.0%
Sex	Male	103	59.5%	32	57.1%
	Female	70	40.5%	24	42.9%
	Total	173	100.0%	56	100.0%
Race/ethnicity	White, not Hispanic	97	69.3%	39	83.0%
	Hispanic or a race other than White	43	30.7%	8	17.0%
	Total	140	100.0%	47	100.0%
Household income	< $15,000	22	11.8%	6	10.7%
	$15,001-$25,000	19	10.7%	5	8.9%
	$25,001-$30,000	8	4.3%	2	3.6%
	$30,001-$35,000	15	8.0%	5	8.9%
	$35,001-$40,000	12	6.4%	5	8.9%
	$40,001-$45,000	14	7.5%	6	10.7%
	$45,001-$50,000	10	5.3%	2	3.6%
	>$50,000	85	46.0%	25	44.6%
	Total	185	100.0%	56	100.0%

Table 2 shows the breakdowns for selected characteristics that are associated with the participants’ experiences as deaf children. Here, considerable differences are noted between the full and target samples; however these differences are due entirely to our selection criteria: a higher percentage of participants from the target sample are reported as having one or both deaf parents (an effect of limiting our target sample to signing families); a higher percentage are reported as having signing in the home (100%); the distribution along the Gallaudet Scale are weighted toward those participants with limited functional hearing (an effect of limiting our sample to those scoring 4 or higher on the Gallaudet Scale); and a higher percentage of parents report that sign language only is the preferred instructional method for communication. A small number of children (3) whose parents reported signing regularly in the home also reported that in the classroom the child was taught using spoken language only and a larger group (18) was reported to be taught using sign supported spoken language. As signing was reported to be used regularly in the home, these children were included in the target sample. It is noteworthy that the percentages of participants with cochlear implants are virtually identical between the full and target samples.

**Table 2:** Comparisons of full EELS and target sample characteristics (deaf-related).

		All survey participants		Target sample	
		N	%	N	%
Is either parent deaf?	Both parents hearing	83	61.5%	28	50.0%
	One or both parents deaf	52	38.5%	28	50.0%
	Total	135	100.0%	56	100.0%
Signing used regularly in the home	No	52	29.1%	--	--
	Yes	127	70.9%	56	100.0%
	Total	179	100.0%	56	100.0%
Functional hearing "My child can hear and understand words when . . .	. . .whispered to across a quiet room	2	1.1%	--	--
	. . .spoken to in a normal voice	19	10.2%	--	--
	. . .shouted to across a quiet room	20	10.8%	--	--
	. . .spoken loudly into my child's better ear	15	8.1%	5	8.9%
	My child can usually tell one kind of noise from another	7	3.8%	2	3.6%
	My child can usually hear loud noises	49	26.3%	21	37.5%
	My child cannot hear anything at all	74	39.8%	28	50.0%
	Total	186	100.0%	56	100.0%
CI reported in any of the three waves	Yes	51	28.2%	16	28.6%
	No	130	71.8%	40	71.4%
	Total	181	100.0%	56	100.0%
Communication used in the classroom	Spoken language only	27	14.9%	3	5.5%
	Sign language only	77	42.5%	33	60.0%
	Sign supported spoken language	70	38.7%	18	32.7%
	Spoken languages with cues	7	3.5%	1	1.8%
	Total	181	100.0%	55	100.0%

As a separate examination of the representativeness of our sample, Table 3 shows the proportional breakdowns for the 37,829 deaf and hard of hearing children comprising the 2009–2010 Annual Survey of Deaf and Hard of Hearing Children and Youth, conducted by the Gallaudet Research Institute [43]. (2009–2010 is the final year that national summaries are available. While it has been nearly 10 years since these data were collected, the summary is nonetheless appropriate as a comparison for the EELS study, as 2010 was also the first year of EELS data collection). We note large discrepancies when comparing the target sample with the national data base, but, again, the differences are due to intentional efforts to focus on a specific more homogeneous sample. The Annual Survey captured the full national population of both deaf and hard of hearing children in the United States receiving special services. The presence of large numbers of hard of hearing children (over 60% are in the “less-than-severe’ category) impacts many of the other characteristics studied. For example, the 15% reported with cochlear implants is misleading, given that children with milder levels of loss are ineligible for implantation.

**Table 3:** National statistics from the Annual Survey of Deaf and Hearing Children and Youth 2009–2010 school year, total N = 37,828.

Sex	% Female	45.8%
Race/Ethnicity	% White and not Hispanic	46.6%
SES	% Economically Disadvantaged	38.2%
Parental hearing status	% Both parents hearing	77.0%
Hearing level	% in the Severe to Profound range	39.8%
Cochlear Implant	% with implant	15.0%
Communication in the classroom	% Spoken language only % Sign language only % Sign supported spoken language % Spoken language with cues	53.0% 27.4% 12.1% 5.0%
Sign use in the home	% Family members regularly sign	23%

Given the importance of age in a developmental study, the design of EELS entailed the specification of Cohorts, defined by the age of each participant, in years as described below. Some of the tables presented in our analysis, employ the Cohort variable as a means for providing a picture of change as the participants developed from year to year. We also presented data for each year of age collapsed over the cohorts reflecting total outcomes for children in the target sample at ages three through eight. In our modeling efforts, we were interested in a finer grain assessment of the participants’ ages, and include months of age at the time of Wave 1 assessments (hereafter referred to as Age). For those with imputed Wave 1 scores, we subtracted 12 from their months of age at the time of testing in Wave 2. Table 4 presents the means and standard deviations of Age for each of the study’s cohorts. The means, all positioned near the center of the range of age within each cohort (e.g., the mean age for C1 was near the midpoint of age three), reveal that that the ages (in months) are fairly well distributed within each cohort. We note that C4 is an exception, with a mean age in months close to 73, near the lower end of the age range for six-year-olds. This resulted from the fact that all participants were between the ages of 3 and 5 when they were recruited into the study; these individuals had already turned six in Wave 1 at the time of their first assessments.

**Table 4:** Mean age (in months) within each cohort at the time of testing in Wave 1.

	C1	C2	C3	C4
Mean	41.87	53.81	65.89	73.83
SD	3.00	3.80	3.30	1.83
Count	15	16	19	6

### Measures and variables selected for analysis

Specific measures extracted from the EELS dataset for the current analysis are described below.

#### Letter and Word Identification Knowledge (LWI)

LWI was measured with the Letter-Word Identification subtest of the Woodcock Johnson, Third Edition Normative Update (NU) Tests of Achievement (WJ-III) [44]. At the earliest stages, this task involves simple upper and lower case letter recognition and naming. As the task progresses, the child must identify simple one syllable consonant-vowel-consonant words (e.g., car) and then words of increasing length and complexity. Instructions were provided and the children responded in their preferred communication (signs and/or speech). To address the research questions, analyses of both W scores and standard scores from the LWI test were included in the study design. W scores represent a transformation of raw scores, which can vary in intervals between scores, to equal interval scores [45]. This allows the comparison of scores across ages, enabling the tracking of progress across time as the changes in W scores represent growth (or decline) in the skill being measured. This contrasts with changes in standard scores, which reflect changes in the child’s performance relative the performances observed in the normative sample. If a child improves their skills at a rate consistent with that of the normative sample, the standard score will remain the same. If they make skill gains at a rate below that of their normative peers the standard score will decline, while rates greater than the normative gains will result in increased standard scores. Thus, W score changes reflect absolute skill growth, while standard score changes reflect growth relative to the normative population. The normative population of the WJ-III included 8,818 individuals from a nationwide sample designed to be representative of the United States population using a stratified sampling design taking factors including census region, community size, sex, race, and ethnicity into consideration [46, 47]. Thus, while the normative sample may not reflect the performances achieved by deaf students, it was representative of the general population of the United States.

Confirmatory factor analysis by Lederberg and colleagues found that WJ-III Letter-Word Identification, Passage Comprehension, and Reading Fluency all loaded heavily (.89 to .97) on a latent Reading factor for three groups of American deaf children using signs, spoken language, or bimodal communication (both signs and speech) [27]. Letter-Word Identification produced the highest loadings (.96 to .97) for all three groups. Similarly, Harris and colleagues found that single word reading (analogous to the word identification portion of Letter-Word Identification) correlated highly (.74) with a reading comprehension for a sample of British children with severe to profound hearing loss [17]. Adolph and colleagues studied a sample of American children without reported hearing loss and found that while the correlation declined to. 36 for eighth grade reading comprehension, Kindergarten Letter Identification (limited to the letter reading portion of Letter-Word Identification) represented one of the two strongest correlations (.61) with second grade reading comprehension [48]. Thus, for both deaf and hearing populations, letter and word identification skills appear to be related to early reading comprehension skills.

#### ASL receptive skills

The American Sign Language Receptive Skills Test (ASL-RST) [49] was administered to participants in each of the three waves of data collection. This test was designed to measure reception of linguistically accurate ASL sentences presented via DVD on a computer and psychometric evidence has supported its use as a measure of ASL receptive skills for pre-school aged children [50]. Each item is presented via a video clip of a signed sentence, after which the child must determine which of four pictures best represents the meaning of the sentence. The length and complexity of the sentences increase over the course of the test. While age-based standard scores are available for older children, these were not yet available for younger children. Thus, raw scores from the Wave 2 administration of the ASL-RST were used in the current analysis. Scores from Wave 2 were analyzed as while we were interested in exploring the predictive relationships between early language skills and literacy development, there were floor effects for the 3-year-old children from Wave 1. Thus, use of Wave 2 data allowed for a wider range of performances to be reflected in the analyses.

#### Cohort

The EELS project employed a cross-sequential design, incorporating both cross sectional and longitudinal elements in its analysis plan. Three cohorts, comprised of children at three different age groups were recruited as participants. These cohorts were 3 (C1), 4 (C2), and 5 (C3) years old in the first wave of the study. Because a small number of participants had turned 6 at the time of their first assessments, we have created a fourth cohort (C4) for these participants. A more detailed description of the age distributions within the sample is provided below.

#### Age

Through the cross-sequential design, it was possible to look at participants at each age (3 through 8) as well as by cohort. Both C1 and C2 included 4-year-old children, and all three cohorts included 5-year-olds. Thus, the outcomes can be considered based on the variables cohort, which separates each group based on age at first assessment, or age, collapsing across the cohorts. For the analyses, the child’s age in months at the time of assessment during Wave 1 will be referred to as Age. Other references to the child’s age (such as means and standard deviations for children at age 4 or 5) will be referred to using lower case.

#### Parental hearing/deaf status

On separate questions, respondents were asked to report on the hearing/deaf status of the participants’ mother and father. The four response options were “*Deaf”*, *“Hard of Hearing”*, *“Hearing”*, *and “Data not Available”*. For analysis, participants were dichotomized by whether they had “*One or Both Deaf Parents”* or “*Both hearing or hard of hearing parents*.*”* This dichotomization yielded subgroups defined by whether there was a deaf parent in the home. Throughout the analysis the variable name “Deaf Parent” refers to this variable.

## Results

### Question 1

The following results were obtained from analyses addressing the question, “What are the trajectories of growth in letter and word identification skills over three years among signing deaf children who were between the ages of 3 and 6 in Wave 1?”.

#### W scores

To examine the levels of performance on the Letter Word Identification (LWI) task and the trajectories of growth over the three years of data collection, we computed means and standard deviations of the LWI W scores by cohort, age, and wave, explored the distributions of these scores by age, wave, and cohort, and examined individual trajectories of each participant as a basis for the calculation of intercept and slope scores for each child [51].

The means and standard deviations of LWI W scores and the number of participants for each subgroup are presented in Table 5. The first three rows (identified by Waves) contain the scores for each cohort across the three waves of the study. To facilitate interpretation of the impact of age on W score performance the statistics are presented so that the ages are aligned within each column of the table. Means and standard deviations aggregated by age across cohorts are presented in the bottom row of each age column. The results demonstrate that each cohort gained in performance level across the three years of testing. A visual inspection of the trajectories for each cohort indicates that while there is significant variability among the curves, the general trend is for positive growth. C1 (three years of age in Wave 1) gained 47 W scale points on average over the three years of the study; C2 (four years of age in Wave 1) gained 62 points; and C3 (five years of age in Wave 1) gained 60 points. While this growth would appear to be greater for the C2 and C3, it should be noted that greater gains are expected for children once they enter formal K-12 education. The variability in the outcomes also appears to increase as the children enter Kindergarten. The standard deviation for C1 (three-year-olds) in Wave 1was 29.77; the standard deviation for C3 at age seven in Wave 3 was 40.44.

**Table 5:** Means, standard deviations, and sample sizes for Letter-Word Identification W Scores for the target sample.

LWI W Scores-Target	Age					
Mean (SD) N	3	4	5	6	7	8
Wave 1	323.27 (29.77) 15 C1	350.50 (41.64) 16 C2	364.79 (28.59) 19 C3	376.17 (39.29) 6 C4		
Wave 2		350.87 (19.88) 15 C1	381.44 (34.06) 16 C2	393.26 (37.09) 19 C3	407.00 (42.73) 6 C4	
Wave 3			370.33 (25.78) 15 C1	412.00 (49.22) 16 C2	425.26 (40.40) 19 C3	425.17 (44.18) 6 C4
Performance by age	323.27 (29.77) 15	350.67 (32.43)31	371.78 (29.92) 50	398.07 (43.37) 41	420.88 (40.84) 25	425.17 (44.18) 6

C1: Cohort 1 (3 years old in the first wave of data collection); C2: Cohort 2 (4 years old in the first wave); C3: Cohort 3 (5 years old in the first wave); C4: Cohort 4 (6ix years old in the first wave)

The outcomes for each age were collapsed across the cohorts, as seen in the bottom row in Table 5. The target sample as a whole made gains of 20 to 28 points per year through age seven. The small difference between the outcomes for ages seven and eight should not be over interpreted as the age eight group consisted solely of C4 (n = 6), whose scores were consistently lower than those of other cohorts within each wave. Overall, there was a 75 point gain in mean W scores between the assessments at age three and those at age eight. Standard deviations remained consistent through age five, but increased dramatically beginning at age six. This reiterates the increased variability in outcomes as the children entered the educational setting.

**Fig 2:**
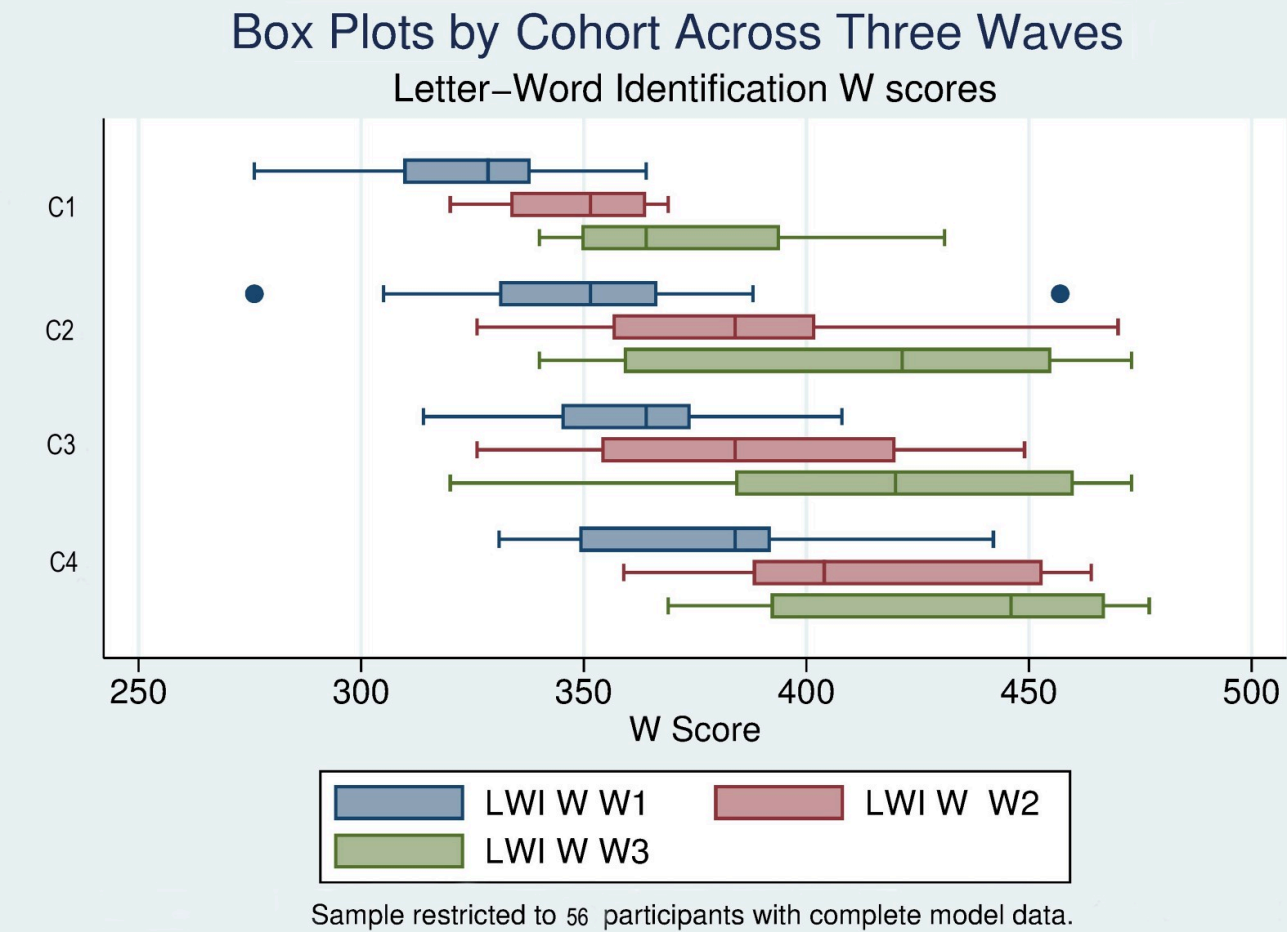
Box plots of Letter Word Identification W scores by cohort across wave.

The distributional analysis is presented in Fig 2. The increases in median performance levels across the waves for each cohort, as well as the increases in variability of scores are clearly in evidence. While the median scores increased across the waves for each group, a number of children in each group demonstrated more limited skills.

**Fig 3:**
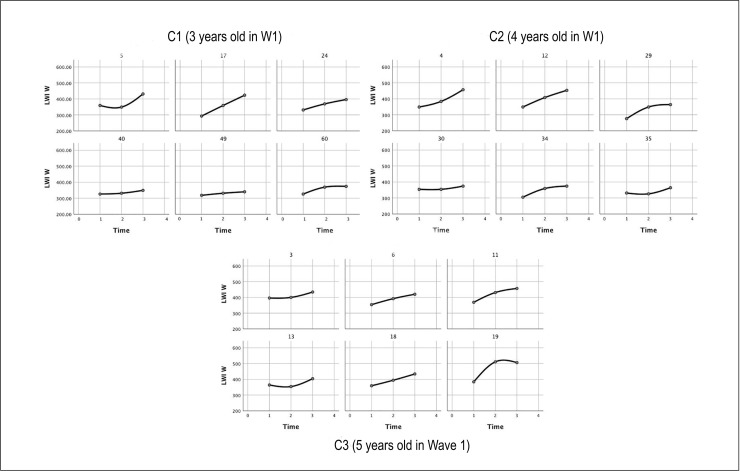
Individual spline curves of participant W scores for the three cohorts across the three waves.

Between-participant variability is further apparent in the sample individual spline curves for the participants (Fig 3), which demonstrate that some of the children achieved relatively steady growth over the years, while others made more limited gains. Those with the lowest initial skills appeared to demonstrate the most limited growth, particularly in the older cohorts.

#### Standard scores

In order to examine the performance of deaf children in the context of the published standardized norms for the test, we followed the same analytical strategy as was presented for the W scale: present the table of means and standard deviations by cohort across waves and ages collapsed across cohorts, evaluate the distributional characteristics through inspection of box plots, and examine the individual growth curves for a small number of participants.

Within the national norming sample, standard scores for the WJ-III are created with a mean of 100 and a standard deviation of 15. Participants whose standard score is equal to 100 are performing comparable to the mean level of performance of individuals in the norming sample who are the same age. A four-year-old child who has a standard score of 115 would have a level of performance equivalent to one standard deviation above the mean of four-year-old participants from the norming sample.

Table 6 reports the mean standard scores by cohort across the three waves of the study and collapsed across the cohorts by age. As can be seen from Table 6, each cohort except C4 scores above the national means at younger ages, but then drop precipitously as they enter elementary school. Participants in C1 and C2 from the target sample (three and four years of age in Wave 1, respectively) stay above 100 throughout the three years of the study. Participants in C3 (age 5 in Wave 1) drop from 103.47 in Wave 1 to 95.84 in Wave 2 and 92.05 in Wave 3 (when they are seven years of age).

**Table 6:** Means, standard deviations, and sample sizes for Letter-Word Identification standard scores for the target sample.

LWI Standard Scores-Target	Age					
Mean (SD) N	3	4	5	6	7	8
Wave 1	105.67 (15.29) 15 C1	108.94 (20.4) 16 C2	103.47 (13.28) 19 C3	95.17 (16.24) 6 C4		
Wave 2		109.20 (8.4) 15 C1	108.00 (14.81) 16 C2	95.84 (13.09) 19 C3	89.0 (17.98) 6 C4	
Wave 3			104.27 (10.12) 15 C1	103.69 (22.54)16 C2	92.05 (14.40) 19 C3	82.00 (18.34) 6 C4
Performance by age	105.67 (15.29) 15	109.06 (15.53) 31	105.16 (12.85) 50	98.81 (17.78) 41	91.32 (14.99) 25	82.00 (18.34) 6

C1: Cohort 1 (3 years old in the first wave of data collection); C2: Cohort 2 (4 years old in the first wave); C3: Cohort 3 (5 years old in the first wave); C4: Cohort 4 (6ix years old in the first wave)

The data collapsed across cohorts representing the groups of children at ages three through eight across the study also reflected this pattern of means higher than the normative mean for ages three through five, averaging one- to two-thirds of a standard deviation above the test mean. By age 6 the mean declined to slightly below the normative mean, and by age seven it was nearly two thirds of a standard deviation below the normative mean. The eight year olds scored more than one standard deviation below the mean. While again this may be an artifact of the small sample in C4, this represented a decline of nearly one standard deviation compared to their performances in Wave 1. The standard deviations were consistent with the normative standard deviation of 15.

We reiterate that (as indicated in the W score analysis) these drops in standard score performance do not indicate a loss in skill level; rather they indicate an increasing lag between the average levels of performance between the target sample and the average levels of performance of a national sample of hearing children of the same age.

**Fig 4:**
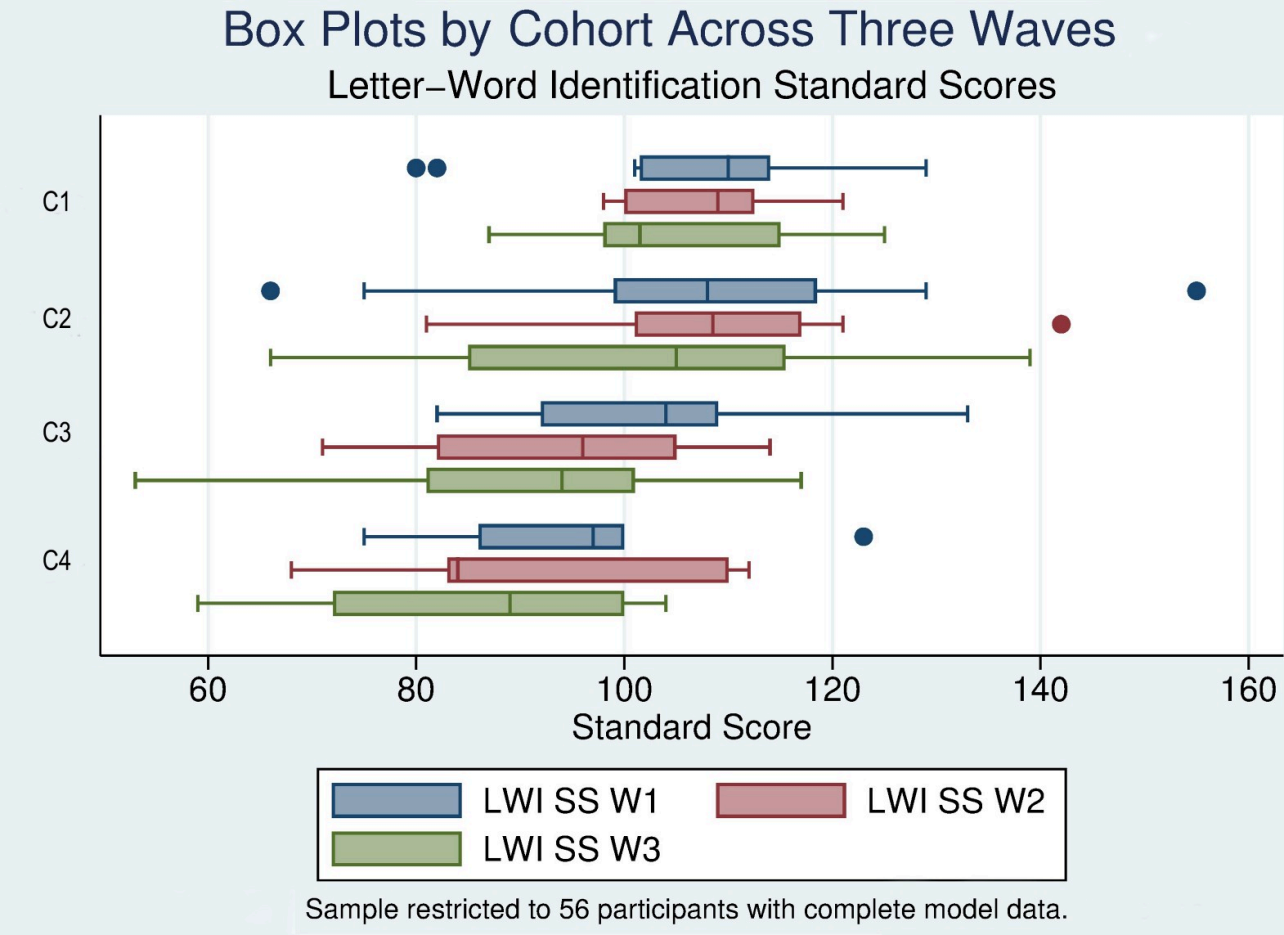
Box plots of Letter Word Identification standard scores by cohort across waves.

The distributional characteristics of the standard scores for the study cohorts across waves are presented in Fig 4. These plots show the declines in median level standard score performance among the older children, and the increase in variability of standard score performance as the participants grow older. As would be expected for children achieving age appropriate growth in skills, the median standard scores of the children in C1 and C2 remain consistent across Waves 1 and 2; however, consistent with the declines seen in each wave for C3 and C4, they decline in the final wave of the study.

**Fig 5:**
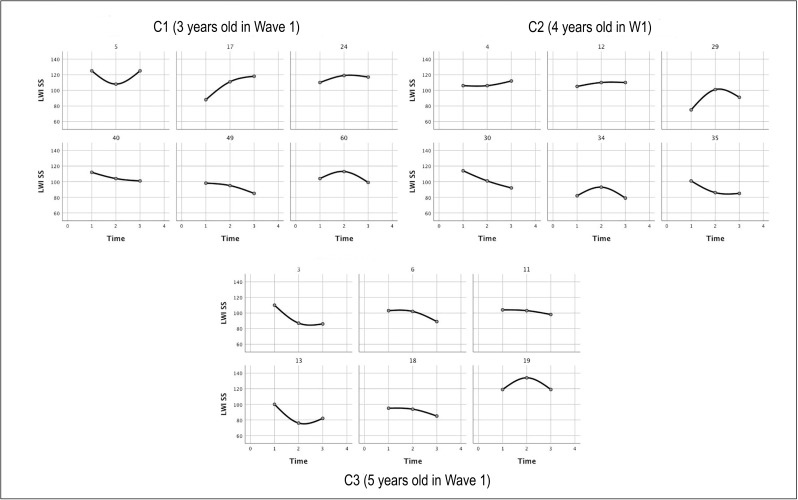
Individual spline curves of participant standard scores for the three cohorts across the three waves.

The individual spline curves (Fig 5) again display the wide variation in trajectories among the participants, with some participants’ scores remaining relatively stable relative to their hearing peers over the years, while others declined significantly, reflecting a lack of gains comparable to hearing peers rather than loss of skills.

### Question 2

The following results were obtained from analyses addressing the question, “To what extent do the presence of a deaf parent in the home and the level of American Sign Language skill (uniquely and in combination) contribute to the overall level and to the rate of growth in letter word identification?”

Using the regressions performed on each participant (a sample of individual growth curves are displayed above in Figs 3 and 5), we calculated intercepts and slopes for each participant on both the W scores and the standard scores. The means and standard deviations of these new variables are presented in Table 7 for the 56 participants in our target sample that will be the basis of our modeling analysis.

**Table 7:** Means and standard deviations of intercepts and slopes of Letter-Word Identification W and standard scores for the target sample.

Target sample	LWI W intercept	LWI W slope	LWI SS intercept	LWI SS slope
Mean	323.24	27.97	108.65	-3.58
Standard Deviation	39.61	13.43	17.75	6.42
N	56	56	56	56

To fully answer Question 2, we constructed 4 hierarchical regression models, one for each of the metrics presented in Table 7. In each model, we entered Age (in months at the time of the first assessment) and, for the slope measures only, the Wave 1 LWI scores on the first step to assess the impact of age and initial levels of performance on the metric being evaluated. In step 2, we added Deaf Parent to assess the impact of having one or both deaf parents in the home) on the metric, controlling for age. In the final step, we added the Wave 2 ASL-RST score. This final model gauges the impact of ASL receptive skills, controlling for the previously included predictor variables. In each model, we present the adjusted R-square values rather than the unadjusted values. This provides a more conservative estimate of the predictability of each model, and was chosen because of our relatively small sample size and the fact that we retained nonsignificant coefficients in the models presented.

The first model, presented in Table 8, shows the results of the hierarchical analysis for the LWI W score intercepts. The intercepts represent the predicted starting points for each participant at the start of the time sequence. In step 1, Age is highly significant as a predictor of LWI W score intercept. On average, each month of age yields a 1.64 point increase in intercept value. The standardized coefficient is a strong .474, and the Adjusted R-square is .21. Adding Deaf Parent in step 2, increases the Adjusted R-square by 3.6 percentage points to .246; the coefficient for Deaf Parent indicates a 17.33 increase in points to the W score intercept when a deaf parent is present. The t-value for the coefficient carries a probability of .065. Adding ASL-RST scores on step 3 changes the complexion of the model dramatically. The coefficients for Age and Deaf Parent drop to a level that is not significant, while the coefficient for ASL-RST carries a strong standardized coefficient (.470) and the Adjusted R-square model after step 3 is .382 represents an increase of 13.6 percentage points over step 2. The results point to the importance of ASL skills, and suggest that the advantages of having a deaf parent lie in its impact on ASL skills.

**Table 8:** Regressions predicting individual intercepts for LWI W scores.

	Age in months at Wave 1	Adding deaf parents	Adding ASL receptive skills
Age in months at time of W1	1.624*** (.474)	1.626*** (.475)	0.758 (.221)
	(3.956, *p <* .*001*)	(4.054 *p <* .*001*)	(1.730, *p =* .*089*)
Participant has one or both deaf parents		17.330 (.221)	7.719 (.098)
		(1.885, *p =* .*065*)	(.882, *p =* .*382*)
ASL receptive skills			1.630*** (.470)
			(3.553, *p =* .*001*)
Observations	56	56	56
Adjusted *R*^2^	.210	.246	.382

Standardized coefficients in parentheses

*t* statistics and probabilities in parentheses

* *p* < 0.05, ** *p* < 0.01

*** *p* < 0.001

The second model, presented in Table 9, shows the impact of our three-tiered model on LWI W score slopes. In the slope models, we have added LWI Wave 1 W scores as a covariate in step 1 of the analysis, given our observation that rates of growth (slopes) may co-vary with initial levels of performance. Step 1 of this model reveals very little in the way of predictability. Neither Age nor LWI Wave 1 performance predicts the individual rates of change in LWI W scores, and the Adjusted R-square is less than 1%. Adding Deaf Parent does not add to the model. The Adjusted R-square remains under 1%. In step 3, adding ASL receptive skills elevates the Adjusted R-square to over 9%, and the standardized coefficient for ASL receptive skills is over 0.5. While none of the other independent variables contribute to the individual rates of growth in LWI, the results suggest that individuals with higher ASL skills do, in fact, have steeper developmental slopes, controlling for Age, initial levels of LWI performance, and having a deaf parent.

**Table 9:** Regressions predicting individual slopes for LWI W scores.

	Age in months at Wave 1 plus initial LWI score level	Adding deaf parents	Adding ASL receptive skills
Age in months at time of W1	.236 (.203)	.213 (.184)	.078 (.068)
	(1.267, *p =* .*211*)	(1.136, *p =* .*261*)	(-0.422, *p =* .*675*)
LWI W score at Wave 1	-.057 (-.161)	-.044 (-.126)	-.151* (-.430)
	(-1.005, *p =* .*319)*	(-.764, *p =* .*448)*	(-2.201, *p =* .*032)*
Participant has one or both deaf parents		-3.598 (-.135)	-5.689 (-.214)
		(-.975, *p =* .*334*)	(-1.583, *p =* .*120*)
ASL receptive skills			0.611* (.519)
			(2.597, *p =* .*012*)
Observations	56	56	56
Adjusted *R*^2^	-.005	-.005	0.095

Standardized coefficients in parentheses

*t* statistics and probabilities in parentheses

* *p* < 0.05

** *p* < 0.01, *** *p* < 0.001

The third model, presented in Table 10, shows the results for LWI standard score intercepts. In Step 1, Age does not predict the standard score intercepts (Adjusted R-square = -.017). Adding Deaf Parent increases the Adjusted R-square to over 5%, and the coefficient for Deaf Parent has a t-statistic that carries a p-value of .031, suggesting that having a deaf parent may contribute to initial LWI standard score performance. However, after step 3, the significance of having a deaf parent decreases, as ASL skill enters the model, and the Adjusted R-square increases to over 16% (an increase of over 11%). Here again, the addition of ASL skills into the model, renders the impact of having a deaf parent nonsignificant, indicating that the benefits of having a deaf parent relate to its impact on ASL skills. Importantly, ASL skills have a strong positive association with the rate of growth.

**Table 10:** Regressions predicting individual intercepts for LWI standard scores.

	Age in months at Wave 1	Adding deaf parents	Adding ASL receptive skills
Age in months at time of W1	-.057 (-.037)	-.056(-.036)	-.417 (-.271)
	(-0.275, *p =* .*784*)	(-.278, *p =* .*782*)	(-1.824, *p =* .*074*)
Participant has one or both deaf parents		10.222* (.291)	6.230 (.177)
		(2.12, *p =* .*031*)	(1.364. *p =* .*178*)
ASL receptive skills			0.677* (.436)
			(2.828, *p =* .*007*)
Observations	56	56	56
Adjusted *R*^2^	-.017	.051	.162

Standardized coefficients in parentheses

*t* statistics and probabilities in parentheses

* *p* < 0.05

** *p* < 0.01, *** *p* < 0.001

The final model, presented in Table 11, shows the results for LWI standard score slopes. We noted above, in Table 8, that the mean slopes for the LWI standard scores is negative, indicating that, on average, participants, when evaluated against norms developed with hearing children, lose ground over time, starting at ages three and four above the national norms, and ending, by age eight below the national norms. The analysis shown in Table 11 reveals a complex set of relationships among the variables Age, LWI Wave 1 performance, Deaf Parent, and ASL skills. After Step1, both Age and initial levels of performance show significant negative relationships with growth rates in scale score performance. Older children, and children who begin with higher levels of performance show declines in standard score performance. The Adjusted R-square after step 1 is over 19%. Adding Deaf Parent in Step 2 does not add significantly to the explanatory impact of the model. However, in Step 3, after ASL skill is entered into the model, all variables become significant and the Adjusted R-square jumps to almost 35%. Interestingly, Deaf Parent, along with Age and Initial LWI performance, significantly contribute to the negative standard score slopes. At the same time, ASL receptive skill shows a strong positive relationship to LWI standard score slopes, controlling for the other predictors (standardized coefficient = .562 and a t statistic carrying a p-value of .001), suggesting that ASL skill may help to mitigate the downward trend in standard score performance over time.

**Table 11:** Regressions predicting individual slopes for LWI standard scores.

	Age in months at Wave 1 plus initial LWI score level	Adding deaf parents	Adding ASL receptive skills
Age in months at time of W1	-.217**(-.392)	-.215** (-.387)	-.400*** (-.722)
	(-3.204, *p =* .*002*)	(-3.181, *p =* .*002*)	(-4.989, *p <* .*001)*)
LWI standard score at Wave 1	-.124*(-.318)	-.108* (-.278)	-.201***(-.516)
	(-2.601, *p =* .*012)*	(-2.214, *p =* .*031)*	(-3.919, *p <* .*001)*
Participant has one or both deaf parents		-2.011 (-.158)	-3.119*(-.245)
		(-1.270, *p =* .*210*)	(-2.130, *p =* .*038*)
ASL receptive skills			.316*** (.562)
			(3.561, *p =* .*001*)
Observations	56	56	56
Adjusted *R*^2^	.192	.201	.348

Standardized coefficients in parentheses

*t* statistics and probabilities in parentheses

* *p* < 0.05

** *p* < 0.01

*** *p* < 0.001

## Discussion

This paper has presented an analysis of a subset of longitudinal data, collected over a three-year period on preschool-aged deaf children making the transition to elementary school. The analyses are based on the subsample of signing families with deaf children who are reported to have limited ability to hear and understand speech and examine the impact of early visual language on emergent literacy for these children.

The current paper focuses on the trajectories of growth in the specific emergent literacy skill of Letter Word Identification. As can be seen from Table 7, the participants gain about 28 W score points per year. This suggests that as a whole the children in this study made gains in letter and word identification skills over the three years studied. However, as seen in Table 5 and Fig 3, these gains varied depending on the age at first assessment. Furthermore, the variability of the outcomes increased in the older cohorts, suggesting that some children made higher levels of gains, while others made quite limited gains. While the observed gains are encouraging, this analysis does not indicate whether the children studied are making the degree of progress needed to achieve levels of print literacy that will ensure adequate skills to function in the later academic and employment settings. To this end, the performances were also analyzed using standard scores based on the WJ-III norming sample.

Initial early literacy skills and the changes in these skills reflected in standard scores over the years were calculated, yielding data which indicated that the young deaf children studied initially averaged early literacy skills above the mean for typical hearing peers. As seen in the W scores, they made annual gains; however, on average these gains did not keep pace with the skill growth of the normative population, resulting in a decline of about 3.5 standard score points per year for the target sample. The changes in standard score outcomes varied depending on the age at first assessment. While the standard scores of C1 and C2 (ages three and four in Wave 1) were relatively stable through age five, standard scores of the older participants demonstrated substantial declines starting in Wave 2. This suggests that while C3 and C4 continued to increase their skills, the skill development of their hearing peers who were also attending early elementary school was greater despite the additional supports typically provided deaf students in early intervention programs and elementary education settings. While it is not clear why the standard scores of the older students declined, possible explanations include lack of adequate early language skills and access to communication in the classroom, and academic environments which do not meet the educational needs of deaf students. This suggests that the educational needs of young deaf children require further investigation in order to ensure that the early gains are maintained as they move on to kindergarten through 12^th^ grade.

Rates of growth over the three years of the study were calculated, using time as the independent measure, creating separate regressions for all participants. In addition to the ongoing growth of skills despite declines in scores relative to hearing norms, high levels of variability were observed among the participants which increased with the participants’ age. Thus, questions remain as to what factors contribute to this variability and specifically, what factors support the stronger skill development of those participants whose skill development best approximates that of their hearing peers. We note that the larger EELS dataset from which the current sample was drawn contains a wealth of information regarding school and home factors that may impact learning and print literacy development. These factors will be examined in future papers.

Two factors have been identified as being potential sources of better literacy skill development: having deaf parents and having better early language skills. Thus, the influences of these two factors were investigated further. Analyses were performed to answer the question that has been pervasive in the in the field of deaf education: *Does having a deaf parent improve the trajectories of literacy growth*? The current outcomes were consistent with the work of Novogrodsky and colleagues, which found that deaf children with hearing parents developed ASL skills on a similar, although delayed, trajectory to children of deaf parents, and that ASL skills accounted for the differences seen in reading outcomes between the two groups [33, 34]. The regression analyses suggest that having a deaf parent does provide a relative benefit, but that when the contribution of ASL skills is added to the model, the Deaf Parent variable no longer provides a significant contribution. This was true for both initial performance on the letter word identification task and the growth in skills over time. Thus, the presence of deaf parents in the home alone does not account for the better skills observed for some of the signing deaf children studied. Instead, the benefit is attributable to greater ASL skills reflecting the presence of a strong first language.

Based upon a previously published cross-sectional study that found strong statistical effects of ASL Receptive Skill (modeled as continuous scale level variables) on a young child’s ability to write letters of the alphabet at a single point in time [37], we hypothesized that visual language skills would also predict steeper learning slopes, would generalize to more complicated reading tasks such as Letter Word Identification, and would be in evidence even when controlled for initial performance levels. The hierarchical analysis model performed to investigate the effects of various factors on early literacy development confirmed this hypothesis. Of the initial score at Wave 1, Deaf Parents, and ASL receptive skills, only the initial performance and ASL skills provided significant contributions to the model for the W score and only ASL receptive skills produced a positive contribution for the standard scores, again supporting the contention that ASL skills rather than parental hearing status was the driving factor for better print literacy outcomes. This suggests that while deaf parents have an advantage in the ability to provide early, consistent ASL modeling for their children, hearing parents who provide early, consistent ASL exposure can also enable their children to develop ASL skills which can support their print literacy development. Parental modeling of ASL may be supported by exposing the child to adults (and peers) with well-developed ASL skills.

Discovering these effects with scaled visual language tasks is a major finding of the current study. Growth in literacy is predicted by previously acquired language skill (including a visual language skill), and not by the mere exposure to sign language through a deaf (or hearing) parent. These findings are consistent with the previously cited literature and support the contention of Hall and colleagues that development of a strong first language, which can be achieved through early access to a linguistically accurate natural signed language, supports the development of deaf children [52]. Too often, researchers, parents, and educators hear simplistic claims like, “exposure to sign language will make your child read better”. To be sure, early exposure to language (spoken or signed) is a necessary condition for literacy development, but it is not sufficient. Additional research should seek to articulate the conditions necessary for language development in the home. For example, what roles do social factors and social contingency [53] play in language learning and later literacy acquisition? What are the mechanisms that aid in the transfer of visual language phonological skills to the processing of the orthographical regularities of letters on a page? What role do factors such as fingerspelling and fingerspelling phonology, studied by Lederberg and colleagues [27], play in bridging ASL to reading?

The younger participants’ initial letter word identification skills averaged above the normative mean. One explanation of this is fact that young deaf children typically receive early intervention services which focus on language and early literacy skill development. All of the children in this study were enrolled in early intervention programs prior to entering kindergarten. This enrichment, which typically focuses on letter and early word recognition skills, appears to provide deaf students with an initial advantage on the measure of early literacy used, which also focuses on these skills. Once the deaf students’ hearing peers start kindergarten and begin receiving educational services similar to those of the deaf children, their skill development outpaces that of the deaf students and this advantage appears to be lost. Thus, the advanced early standard scores decline as the children progress through the early elementary school years despite continued skill development. The results of the hierarchical regression suggest that presence of strong language (ASL) skills appears to counteract the relative weakness in literacy development in the deaf children studied. These results are consistent with previous longitudinal studies which found that deaf children’s rates of literacy development declined relative to hearing peers in elementary school, and that, as with early visual language skills, early spoken language skills (e.g., vocabulary, speechreading, letter-sound knowledge) predicted better reading skills [14, 17, 38]. Thus, it appears that underlying language competence, regardless of whether it involves spoken or visual language skills, supports early literacy skill development.

If early ASL skills support early literacy skill development this raises the question as to why skills in a language that differs not only in vocabulary and linguistic structure but also in modality would support the development of literacy skills in English. While this is an area that requires further investigation, some potential mechanisms should be considered.

One possible explanation for the apparent facilitative effects of ASL skills on English literacy development is the fact that having language allows for the accumulation of information. While having more prior knowledge would be expected to better support reading comprehension, where context may be used to enhance comprehension, than basic reading skills, it is still possible that having a broader range of knowledge may facilitate the learning of print vocabulary represented in the measured task. Knowledge about the world facilitates not only reading comprehension through context, it also facilitates signed or spoken language reception, which may allow the child to obtain greater benefit from information presented in the classroom. Additionally, fingerspelling is an integral part of ASL. Since the manual alphabet represents the letters of spoken language being represented, fingerspelling maps directly onto the letters and words represented in letter word identification tasks. Thus, another possible mechanism of the better early reading skills of the deaf children with better ASL skills may be the development of fingerspelling skills which Stone and colleagues reported to have a facilitative effect on reading [54].

A further possible mechanism relates to social skills and school readiness. Previous research suggests that early social competence is facilitated by stronger language skills [55]. Social competence, in turn, contributes to school readiness. Allen and colleagues also identified a relationship between early ASL skills and visual attention, which could also support early reading skill development [55]. Thus, there are multiple possible mechanisms through which early ASL skills could facilitate the development of early literacy skills in deaf children despite differences between English and ASL.

Additional research is needed to clarify the similarities and differences of language environments provided to very young deaf children in their first two or three years of life, a critical period for language development. How does the delay in access to spoken language for deaf children, relative to the at-birth access to visual language, impact the later trajectories of literacy acquisition? Finally, are greater gains to be found among children raised in bilingual and bimodal language environments where both spoken and visual languages are present? Other lines of research (not discussed here) suggest this might be so.

### Limitations and future research

#### Sampling

The sampling strategy used in the current study is both a strength and a limitation. Its strength lies in the fact that we have isolated one homogeneous cluster (deaf children with limited functional hearing who are reported to be receiving sign language in their homes) from a larger, more heterogeneous sample. We noted earlier that previous research on early literacy skills within the population of “deaf and hard of hearing” children are conducted with samples that are both small, heterogeneous with respect to degree of hearing loss and communication methods used in the home, and often over represent children who are hard of hearing and who have access to spoken language. Such studies can erroneously lead to prescriptions for practice that are not valid for all subpopulations such as the one represented here. This strength is also a limitation, as our sample cannot generalize to the broader sample of deaf and hard of hearing children from homes where signing is not used.

As noted in Table 1, our sample underrepresented children from minority backgrounds. One potential reason for this was the complexity of the language in the background surveys administered to parents. While we offered surveys in both Spanish and English print and ASL interviews, language complexity may have also influenced the responses from parents with more limited skills in those languages who did complete the surveys. This could have biased the results. Future research should attend to diversity and the communication needs of the respondents.

#### Missing data

As noted in the Sample Venn Diagrams presented in Fig 1, there was a considerable amount of missing information from the 254 children who participated in the EELS study. Of the 254, only 147 participants had data from both the parents’ background questionnaires and from the battery of assessments. Additionally, not all of these 147 had Letter-Word Identification scores in all three waves of the study. We were able to impute LWI scores for participants who had scores on two of the three assessments and who had valid assessments in Wave 3, but were not able to impute missing family background data for children with Assessment data only. Finally, we eliminated a total of 91 participants from the 147, including children who did not meet the selection criteria for our study, and who had missing data on the independent variables we had selected for our regression modeling.

We note the difficulty in collecting complete data for the study, which was designed to be inclusive of deaf children (with severe to profound hearing losses) from a national population, from different school and program placements, with different language backgrounds, from different races, with different types of assistive listening devices. This was an ambitious enterprise, especially given the combination of factors: 1) a low prevalent population; 2) a diverse population; 3) an extremely young population; and 4) a mobile population requiring individual assessments given each participant at three distinct points in time. While we did not achieve the depth and breadth of sample we had desired, we believe the findings are provocative and will, perhaps, encourage more longitudinal studies of this population to corroborate and expand the results presented here.

#### Use of OLS to estimate growth

Typically, an accurate assessment of growth requires large samples measured at different times (usually more than 3 times, as was the case in the current study), and entails the estimation of growth parameters that includes estimates of measurement quality and estimates of the veracity of the parameters, (e.g., is a linear slope a good representation of an individual’s development over time?). While the current effort does not achieve these standards for longitudinal design, we feel that the analysis presented here has great utility as a first attempt to examine the association between early visual language skills and the rate of literacy acquisition. Indeed, the discovery of strong predictive relationships between measures of early visual language skills and the rate of growth in emerging letter and word identification skills will hopefully lead to the design of more extensive longitudinal studies employing larger, homogeneous samples that will explore these relationships in greater depth, include measures of more complex and higher-order tasks such as reading comprehension, and extend into the middle school years.

## Supporting information

S1 Data SPSS dataset containing study variables. Data are from the Early Education Longitudinal Study subset of deaf children from signing families.
(SAV)

S1 Survey Parents survey from year 1 of study. (PDF)
