# Supplementary material for: Early visual language skills affect the trajectory of literacy gains over a three-year period of time for preschool aged deaf children who experience signing in the home
Source: PLoS One. 2020 Feb 27;15(2):e0229591. doi: 10.1371/journal.pone.0229591 (PMC7046216; doi:10.1371/journal.pone.0229591)

Year one parent survey

SECTION A RESPONDENT INFORMATION

A1. Today's date: Month ________ Day _______ Year ________

A4. Does the child named above currently live with you? ▭ Yes ▭ No

a. If the child does not currently live with you, who does the child currently live with?

▭ Both biological parents
▭ Biological mother
▭ Biological father
▭ In foster care
▭ Legal guardian
▭ Adoptive parent(s)
▭ Other relative (specify________________________)

A5. Your relationship to this child:

▭ Mother ▭ Father ▭ Legal Guardian ▭ Relative (specify: ____________________________)

▭ Adoptive Parent(s) ▭ Other (specify: ____________________________________)

A6. Indicate your hearing status: ▭ Hearing ▭ Hard of Hearing ▭ Deaf

A7. Indicate the hearing status of the following:

Your Spouse or Partner	▭ Hearing	▭ Hard of Hearing	▭ Deaf	
Child's Mother	▭ Hearing	▭ Hard of Hearing	▭ Deaf	▭ Data not available
Child's Father	▭ Hearing	▭ Hard of Hearing	▭ Deaf	▭ Data not available

A8. Do any family members regularly sign to the student in the home?

▭ Yes ▭ No ▭ Data not available

A9. Indicate the language(s) or sign system(s) that are regularly used in the child's **home**. (*Mark all that apply*).

▭ English ▭ Spanish ▭ ASL ▭ Signed English ▭ Other: ____________________________

SECTION B CHILD INFORMATION

B1. Sex: ▭ Male ▭ Female

B2. Child's birthday: ▭▭ ▭▭ ▭▭▭▭
Month Day Year

B3. Choose one or more categories that best describe your child's race.

▭ African American or Black
▭ American Indian or Alaska Native
▭ Asian
▭ Native Hawaiian or other Pacific Islander
▭ White
▭ Don't know

B4. Choose the response that best describes your child's ethnicity: ▭ Hispanic/Latino ▭ Not Hispanic/Latino

B5. Which one of the following communication modes is PRIMARILY used to teach this child?

▭ spoken language only
▭ sign language only
▭ sign supported spoken language
▭ spoken language with cues

- other: ______________________________

B6. Is your child learning to understand or use cued speech? ▭ Yes ▭ No

B7. Is your child learning to understand or use oral speech? ▭ Yes ▭ No

B8. Is your child learning to understand or use sign language? ▭ Yes ▭ No [go to B9]

a. If yes, what form of sign language is your child learning to use?

- ▭ American Sign Language
- ▭ Signed English (sign in English word order)
- ▭ Some other sign language system (specify: ______________________________)
- ▭ Don't know

B9. How old was your child when a hearing loss was first suspected?

▭ at birth ▭ under three years of age → age: ______ ▭ three years of age or older → age: ______

B10. How old was your child when the hearing loss was diagnosed?

▭ at birth ▭ under three years of age → age: ______ ▭ three years of age or older → age: ______

↓

a. If you answered AT BIRTH above, was your child's hearing loss identified through newborn hearing screening at the hospital?

▭ Yes ▭ No ▭ Unsure

B11. Select the statement that best describes your child's ability to hear and understand speech without using hearing aids.

- ▭ My child can usually hear and understand what someone says without seeing the person's face when whispered to across a quiet room.
- ▭ My child can usually hear and understand what someone says without seeing the person's face when spoken to in a normal voice across a quiet room.
- ▭ My child can usually hear and understand what someone says without seeing the person's face when shouted to across a quiet room.
- ▭ My child can usually hear and understand when someone speaks loudly into my child's better ear.
- ▭ My child can usually tell one kind of noise from another.
- ▭ My child can usually hear loud noises.
- ▭ My child can't hear anything at all.

B12. Was your child born 3 or more weeks before he/she was due? ▭ Yes ▭ No ▭ Don't know

B13. How much did your child weigh at birth? Pounds (lbs) ______ ounces (oz) ______
Kilograms ______ ▭ Don't know

B14. As a newborn, did your child stay in the hospital after he/she was born because of medical problems?

▭ No ▭ Yes → If yes, reason(s): ______________________________
▭ Don't know

B15. Other than hearing loss, does your child have a developmental delay or disability?

▭ Yes ▭ No ▭ Don't know

a. If yes, indicate which developmental delay(s) or disability(s) your child has other than his/her hearing impairment:

- ▭ Visual impairment
  - ▭ Low Vision
  - ▭ Legal blindness
- ▭ Developmental delay
- ▭ Learning disability
- ▭ Mental retardation
- ▭ Emotional disturbance
- ▭ Autism
- ▭ Usher syndrome
- ▭ Other health impairment(s)

- Orthopedic impairment
- ADD/ADHD
- Traumatic brain injury
- Specific signed language impairment
- Other: ______________________________

B16. What type of school(s) or program(s) does your child currently attend? (*Mark all that apply*.)

- elementary school
- preschool program in an elementary school
- early childhood or preschool center, or a nursery school
- child care center
- child development center
- home-based services
- other type program/child care (specify: ______________________________)
- don't know

B17. What is your child's current grade level?

- Preschool, full-day
- Preschool, part-day
- Kindergarten, full-day
- Kindergarten, part-day
- First Grade

B18. If your child's program is a residential/boarding school, does your child reside at the school during the week?

- Yes
- No

B19. Does your child have any **hearing** siblings? — Yes — No

B20. Does your child have any **deaf or hard of hearing** siblings? — Yes — No

B21. Has your child ever used a hearing aid? — Yes — No [skip to B.30]

B22. How old was your child when he/she first obtained hearing aid(s)? Age: __________

B23. How many hearing aids did your child start with? — 1 — 2

B24. Does your child still use his/her hearing aid(s)? — Yes — No

If NO, at what age were the hearing aids discontinued? ________ Briefly indicate why: ____________________

____

B25. How many hearing aids does your child currently use? — 1 — 2

B26. What type of hearing aid technology does your child currently use?

- Analog [*go to B28*]
- Digital
- Don't know

B27. If your child uses digital hearing aids, what type of digital hearing aid is used?

- Entry level, economy digital
- Mid-level digital
- Premium, high end digital
- Don't know

B28. What is the manufacturer of your child's current hearing aids?

- Oticon
- Phonak
- Siemens
- Unitron
- Starkey
- GN Resound
- Widex
- Sonovation
- Other (please specify: ____________________)
- Don't know

B29. Does your child use an FM system, coupled with his/her hearing aids, in the classroom?

- Yes
- No
- Don't know

B30. Has your child ever received a cochlear implant (CI)?

- ☐ No (**Skip *to Section D***: *Communication and Language*)
- ☐ Yes **CONTINUE WITH SECTION C**

SECTION C COCHLEAR IMPLANT (CI)

You have indicated that your child has received a cochlear implant (CI). Please answer the questions in this section.

C1. Does your child still use his/her cochlear implant? ☐ Yes ☐ No

If NO, WHEN was CI use discontinued? Year:_______ Briefly indicate why: ______________________________

C2. How old was your child when his/her CI was implanted? ___ Months of age ☐ Don't know

___ Years of age

C3. Does your child use an FM system coupled with his/her implant in the classroom?

☐ Yes ☐ No ☐ Don't know

C4. Does your child use a hearing aid along with the cochlear implant? ☐ Yes ☐ No

C5. Which of the following best represents how your child communicated at home before receiving the cochlear implant? *(Mark only ONE.)*

- ☐ speech only
- ☐ mainly speech
- ☐ more speech than signing
- ☐ roughly equal amounts of signing and speech
- ☐ minimal (if any) spoken words or signs
- ☐ more signed vocabulary than spoken words
- ☐ mainly signing
- ☐ other (*specify)* ______________________________

C6. Which of the following best represents the communication mode used by <u>others</u> in the home when communicating with your child before receiving the cochlear implant? *(Mark only ONE.)*

- ☐ speech only
- ☐ mainly speech
- ☐ more speech than signing
- ☐ roughly equal amounts of signing and speech
- ☐ minimal (if any) spoken words or signs
- ☐ more signed vocabulary than spoken words
- ☐ mainly signing
- ☐ other (*specify)* ______________________________

C7. Which of the following most closely represents the general mode of communication you desired for your child before you considered the cochlear implant? (*Mark only ONE.)*

- ☐ I definitely wanted auditory-oral
- ☐ I was leaning toward sign
- ☐ I wanted a combination of auditory-oral and sign
- ☐ I was leaning toward auditory-oral communication.
- ☐ I definitely wanted sign

C8. Who first suggested the cochlear implant as an option for your child? (*Mark only ONE*.)

- ☐ pediatrician
- ☐ otolaryngologist/ENT
- ☐ other medical professional
- ☐ audiologist
- ☐ teacher
- ☐ family member
- ☐ friend
- ☐ TV program
- ☐ books or other reading material
- ☐ other: ______________________________

C9. Which of the following information *sources* did you use in making the implant decision? *(Mark ALL that apply)* [**In addition, <u>circle</u> the ONE** source of information you think was **most important** in making that decision.]

- ☐ your child
- ☐ adults with CIs
- ☐ ENT/otolaryngologist
- ☐ deaf adult(s)
- ☐ other *(specify)* ______________________________
- ☐ your child's other parent/guardian
- ☐ children with CIs
- ☐ pediatricians
- ☐ videos/DVDs, internet, chat rooms, etc.
- ☐ printed literature
- ☐ parents of CI children
- ☐ audiologists (or other hearing & speech clinicians)

C10. How long after your child's hearing loss was identified did you consider the CI option?

☐ less than 3 months ☐ 3 - 6 months ☐ 7 - 11 months ☐ one year or more

C11. How long did you consider the CI option before deciding to get the CI for your child?

▭ less than 3 months ▭ 3 - 6 months ▭ 7 - 11 months ▭ one year or more

C12. What was your **main reason** for deciding to get a cochlear implant for your child? *(Mark only ONE.)*

▭ child expressed desire for CI
▭ concern for child's positive self-image
▭ convenience in daily activities
▭ ease in development & use of oral spoken language
▭ safety/environmental awareness
▭ other *(explain)* ______________________________

C13. At the time the (initial) CI surgery was performed, how well informed were you regarding:

a. the various *assistive listening devices* (e.g., tactile aids, FM system, amplified telephone, closed captioning) available to your deaf child?

▭ minimally informed ▭ fairly well informed ▭ quite thoroughly informed

b. the various *alternative communication options* (e.g., cued speech, tactile aids, American Sign Language) available to your deaf child?

▭ minimally informed ▭ fairly well informed ▭ quite thoroughly informed

C14. When the implant surgery (initially) was performed, were you *rather unfamiliar, somewhat familiar*, or *very familiar* with various POSSIBLE ***negative outcomes*** of receiving a CI?

	rather unfamiliar	***somewhat familiar***	***very familiar***
auditory/audiological outcomes	▭	▭	▭
medical/health-related outcomes	▭	▭	▭
social outcomes	▭	▭	▭
psychological outcomes	▭	▭	▭
language outcomes	▭	▭	▭

Elaborate, if you wish: ______________________________

C15. When the implant surgery (initially) was performed, were you *rather unfamiliar, somewhat familiar*, or *very familiar* with various POSSIBLE ***positive outcomes*** of receiving a CI?

	rather unfamiliar	***somewhat familiar***	***very familiar***
auditory/audiological outcomes	▭	▭	▭
medical/health-related outcomes	▭	▭	▭
social outcomes	▭	▭	▭
psychological outcomes	▭	▭	▭
language outcomes	▭	▭	▭

(Elaborate, if you wish: ______________________________)

C16. In which ear does your child have a cochlear implant? ▭ Right ▭ Left ▭ Both [Go to C19]

C17. When was your child's (initial) CI surgery performed?

Right Ear (if applicable): ▭▭ Month ▭▭▭▭ Year Left Ear (if applicable): ▭▭ Month ▭▭▭▭ Year

C18. When was your child's CI first stimulated (i.e., date of "hook up")?

Right Ear (if applicable): ▭▭ Month ▭▭▭▭ Year Left Ear (if applicable): ▭▭ Month ▭▭▭▭ Year

C19. Indicate the child's type of cochlear implant: ▭ internal [go to C22] ▭ external [go to C23]

C20. If your child's cochlear implant is internal, what type of equipment did your child receive when implanted? (Please check the box and indicate the ear for each if your child is a bilateral CI recipient.)

				Right Ear	Left Ear
a. Cochlear	☐ N24	☐ Freedom	☐ Nucleus 5	☐	☐
b. Advanced Bionics	☐ Hi Res 90K			☐	☐
c. Med El	☐ Combi 40+	☐ Pulsar	☐ Sonata	☐	☐
d. Don't know	☐				

C21. If your child's cochlear implant is external, what type of equipment did your child receive when implanted? (Please check the box and indicate the ear for each if your child is a bilateral CI recipient.)

					Right Ear	Left Ear
a. Cochlear	☐ Sprint	☐ Esprit 3G	☐ Freedom	☐ Nucleus 5	☐	☐
b. Advanced Bionics	☐ PSP	☐ Auria	☐ Harmony		☐	☐
c. Med El	☐ Tempo+	☐ Opus 1	☐ Opus 2		☐	☐
d. Don't know	☐					

C22. Does your child use a behind-the-ear external CI speech processor? ☐ Yes ☐ No

C23. Does your child use a body-worn CI speech processor? ☐ Yes ☐ No

UNLESS OTHERWISE NOTED, refer to your child's currently-used (or most-recently received) external component or processor, when answering the remaining questions.

C24. During the implant surgery, were the CI electrodes successfully inserted? ☐ Yes ☐ No

a. **If NO**, were you informed, **before the surgery,** of the possibility of a partial insertion? ☐ Yes ☐ No

C25. Are any of the inserted CI electrodes now **in**active–either through hardware failure or voluntary shut off?

☐ Yes ☐ No ☐ Unsure

a. IF **YES**, *indicate why:* ______________________________

C26. In what type of center or facility was the cochlear implant surgery performed?

☐ Private practice ENT
☐ Medical center
☐ Children's hospital
☐ Other: ______________________________

C27. Is your child currently receiving any CI follow-up services (e.g., reprogramming, check ups) from the same implant center where evaluation and surgery occurred?

☐ Yes → How often? ____________

☐ No → Explain the change*:* ______________________________

C28. Indicate the professionals **affiliated with your implant center** who were involved in preparing for and facilitating your child's use of the cochlear implant. *(Mark all that apply.)*

☐ psychologist ☐ surgeon/otolaryngologist ☐ audiologist ☐ speech/language pathologist
☐ educational consultant ☐ social worker ☐ teacher of the deaf
☐ other: ______________________________

C29. Is there **a person** designated to coordinate CI-related services to your child, in or outside of school?

☐ Yes (*indicate person's general job title*) ______________________________ ☐ No

a. If YES, how effective is this liaison? ☐ not effective ☐ mildly effective ☐ very effective

C30. **During the first year of CI use**, how often does (or *did*) your child receive a check up of CI functioning (with or without re-programming)?

☐ less than once a year ☐ once a year ☐ 2 or 3 times/year

▭ 4 or more times/year →→ How many times? ____________

C31. **During the first year of CI use**, how often does (or *did*) your child have his/her CI re-programmed or re-mapped?

▭ less than once a year ▭ once a year ▭ 2 or 3 times/year ▭ 4 or more times/year : How many times? ____________

C32. **After the first year of CI use**, how often does (or *did*) your child receive a check up of CI functioning (with or without re-programming)?

▭ less than once a year ▭ once a year ▭ 2 or 3 times/year ▭ 4 or more times/year: How many times? ____________

C33. **After the first year of CI use**, how often does (or *did*) your child have his/her CI re-programmed or re-mapped?

▭ less than once a year ▭ once a year ▭ 2 or 3 times/year ▭ 4 or more times/year: How many times? ____________

C34. How satisfied were you with the **counseling** you received from your child's implant team **prior to the CI surgery**?

▭ very dissatisfied ▭ dissatisfied ▭ satisfied ▭ very satisfied ▭ I don't recall receiving any

C35. How **generally satisfied are you** with the follow-up/habilitation services you and your child have received (directly or indirectly) **through the implant center**?

▭ very dissatisfied ▭ dissatisfied ▭ satisfied ▭ very satisfied ▭ unsure

C36. For which of the following did your medical insurance provide full or partial financial coverage (with the exception of nominal co-payment.) *(Mark ALL that apply.)*

	Full coverage	***Partial coverage***	***No coverage***	***Not Applicable***
CI re-programing/re-mapping	▭	▭	▭	▭
**Post-implant** speech production therapy	▭	▭	▭	▭
**Post-implant** auditory habilitation training	▭	▭	▭	▭

Other insurance covered accommodations/services *(describe)* ________________________________

C37. **For what period of time** after the initial CI "hook up" did your medical insurance cover **CI habilitation services** such as speech perception training and speech production therapy? *(Select the ONE best-fitting response.)*

▭ none at all ▭ less than 6 months ▭ 6 - 11 months ▭ 12 - 17 months
▭ 18 - 23 months ▭ _______(#) years ▭ other (*specify*) ____________________

C38. Did you *ever* obtain speech communication training for your child, **at your own (i.e., family) expense**?

▭ No *[skip to #30]*
▭ Yes, **for what period of time**? ___ (#) months, *if under 36 months* ___ (#) years, *if at least 3 years*
↓
If YES, why was this "out-of-pocket" training necessary?

▭ appropriate habilitation was not covered under family medical insurance, nor in child's school program
▭ length or frequency of habilitation services covered under medical insurance was inadequate
▭ insurance-covered therapist was not conveniently located to family residence
▭ other (*indicate*: ____)

C39. Did your implant center provide assistance in obtaining appropriate educational supports and services for your child's use of the cochlear implant?

▭ Yes ▭ No

C40. Was the implant center team that arranged and performed your child's implant effective in advising you about CI issues related to:

speech perception? ▭ Yes ▭ No

spoken language production? ▭ Yes ▭ No
the CI device? ▭ Yes ▭ No
educational concerns? ▭ Yes ▭ No
language development? ▭ Yes ▭ No

C41. Considering the period from before the implant surgery, through the habilitation and educational adjustment, and after the surgery, do you feel that you are/were a vital and valued member of your child's CI team?

▭ Yes ▭ No

C42. Was sign communication used with your child **before** getting the CI? ▭ Yes ▭ No *[skip to C44]*

If yes, which of the following statements best characterizes your pattern of sign communication exchanges during the first year or two after the CI was received? *(Mark only ONE.)*

▭ sign communication exchanges were discontinued immediately following CI "hook up"
▭ signing was phased out on a regulated or prepared schedule
▭ your child's use of signing disappeared gradually as auditory-oral communication improved
▭ sign communication continued **as a support** to spoken communication with the CI
▭ sign communication continued **as an alternative** to spoken communication with the CI
▭ other (indicate: ____)

C43. Indicate the total period of time over which your child received any speech training at school or elsewhere:

____ (#) months, *if under 36 months* **or** _______ (#) years, *if at least 3 years* ▭ none

C44. Since the CI "hook up" how many years of any regularly scheduled speech production therapy has your child received at school or elsewhere?

▭ none ▭ less than one year ▭ 1 – 2 years # years __________

a. How would you describe the influence that speech production therapy has had on your child's spoken communication abilities?

▭ Not at all helpful ▭ Somewhat helpful ▭ Very helpful

C45. Since the CI "hook up" how many years of any regularly scheduled listening training has your child received at school or elsewhere?

▭ none ▭ less than one year ▭ 1 – 2 years # years __________

a. How would you describe the influence that listening training has had on your child's spoken communication abilities?

▭ Not at all helpful ▭ Somewhat helpful ▭ Very helpful

C46. How many years of auditory-verbal therapy (AVT) did your child receive before and after receiving the CI?

▭ none ▭ less than one year ▭ 1 – 2 years # years __________

a. How would you describe the influence that auditory-verbal therapy (AVT) has had on your child's spoken communication abilities?

▭ Not at all helpful ▭ Somewhat helpful ▭ Very helpful

C47. How satisfied does your child seem to be with his/her use of the CI?

▭ Very dissatisfied ▭ Dissatisfied ▭ Satisfied ▭ Very satisfied ▭ Unsure

SECTION D COMMUNICATION AND LANGUAGE

D1. Communication (Items redacted due to copyright)	Is not able	Never when needed	Sometimes when needed	Always when needed
		**FREQUENCY**		
[redacted]	☐	☐	☐	☐
[redacted]	☐	☐	☐	☐
[redacted]	☐	☐	☐	☐
[redacted]	☐	☐	☐	☐
[redacted]	☐	☐	☐	☐
[redacted]	☐	☐	☐	☐
[redacted]	☐	☐	☐	☐
[redacted]	☐	☐	☐	☐
[redacted]	☐	☐	☐	☐
[redacted]	☐	☐	☐	☐
[redacted]	☐	☐	☐	☐
[redacted]	☐	☐	☐	☐

[redacted]

[redacted]	Is not able	Never when needed	Sometimes when needed	Always when needed
[redacted]	☐	☐	☐	☐
[redacted]	☐	☐	☐	☐
[redacted]	☐	☐	☐	☐
[redacted]	☐	☐	☐	☐
[redacted]	☐	☐	☐	☐
[redacted]	☐	☐	☐	☐
[redacted]	☐	☐	☐	☐
[redacted]	☐	☐	☐	☐
[redacted]	☐	☐	☐	☐
[redacted]	☐	☐	☐	☐
[redacted]	☐	☐	☐	☐
[redacted]	☐	☐	☐	☐
[redacted]	☐	☐	☐	☐
[redacted]	☐	☐	☐	☐
[redacted]	☐	☐	☐	☐
[redacted]	☐	☐	☐	☐
[redacted]	☐	☐	☐	☐
[redacted]	☐	☐	☐	☐
[redacted]	☐	☐	☐	☐

[redacted]

☐	☐	☐	☐
☐	☐	☐	☐
☐	☐	☐	☐
☐	☐	☐	☐
☐	☐	☐	☐
☐	☐	☐	☐
☐	☐	☐	☐
☐	☐	☐	☐
☐	☐	☐	☐
☐	☐	☐	☐
☐	☐	☐	☐
☐	☐	☐	☐
☐	☐	☐	☐
☐	☐	☐	☐
☐	☐	☐	☐
☐	☐	☐	☐
☐	☐	☐	☐
☐	☐	☐	☐
☐	☐	☐	☐
☐	☐	☐	☐
☐	☐	☐	☐
☐	☐	☐	☐

SECTION E CHILD BEHAVIOR, ACTIVITIES OUTSIDE OF SCHOOL, AND LITERACY

[redacted]

BEHAVIOR FREQUENCY			
Is not able	**Never** when needed	**Sometimes** when needed	**Always** when needed
☐	☐	☐	☐
☐	☐	☐	☐
☐	☐	☐	☐
☐	☐	☐	☐
☐	☐	☐	☐
☐	☐	☐	☐
☐	☐	☐	☐
☐	☐	☐	☐
☐	☐	☐	☐
☐	☐	☐	☐
☐	☐	☐	☐
☐	☐	☐	☐
☐	☐	☐	☐

[redacted]

Is not able	Never when needed	Sometimes when needed	Always when needed

Material from the ABAS-II copyright © 2000, 2003 by Western Psychological Services. Adapted for authorized use in specific scholarly application by T. Allen, Gallaudet University, under limited-use license from the publisher, Western Psychological Services, 12031 Wilshire Boulevard, Los Angeles, California 90025-1251, U.S.A. All rights reserved. No additional reproduction may be made, whether in whole or in part, without the prior, written authorization of Western Psychological Services (rights@wpspublish.com).

Not at Once or 3 – 5 Don’t

	all	twice	times	Daily	know
E5. How often per week does your child look at picture books or other books outside of school?	▭	▭	▭	▭	▭
E6. How often per week does your child read to himself or herself or others outside of school?	▭	▭	▭	▭	▭
E7. How often do you or someone in your family read to your child?	▭	▭	▭	▭	▭
E8. If you have a home computer that your child can use, how often does he/she use the computer?	▭	▭	▭	▭	▭
E9. How often does your child watch educational programs such as *Sesame Street?*	▭	▭	▭	▭	▭
E10. How often does your child engage in letter play (e.g., magnetic letters on the refrigerator)?	▭	▭	▭	▭	▭

E12. In an average week day, indicate approximately how many hours your child does the following:

a. watches captioned TV alone _______ hrs/day
b. watches non-captioned TV alone _______ hrs/day
c. plays with brothers, sisters, or other children in the household _______ hrs/day
d. plays with children from outside the household _______ hrs/day
e. plays alone with no one else in the room or yard _______ hrs/day
f. interacts with household members _______ hrs/day
g. uses a computer to play games _______ hrs/day
h. uses a computer for educational programs/games _______ hrs/day
i. plays on gaming devices (DS, Wii, PS, LMAX, etc) _______ hrs/day
j. uses the VP or phone _______ hrs/day

E13. Do you label items around the house with labels or 'stickies' (e.g., 'stove", "chair", "cupboard", "sink")?

▭ Yes ▭ No

E14. Do you fingerspell words for items around the house? ▭ Yes ▭ No

E15**. If you are using fingerspelling in the home, or if your child's school program is teaching fingerspelling to your child, please indicate your child's level of skill in the following:**	Is not able	**Never** when needed	**Sometimes** when needed	**Always** when needed
Can fingerspell his/her own name	▭	▭	▭	▭
Engages in "fingerbabbling", (i.e., purposeful use of finger/ hand shapes that may not be accurate letters or signs)	▭	▭	▭	▭
Knows and can produce the handshapes of 5 or more letters	▭	▭	▭	▭
Knows and can produce the handshapes of 10 or more letters	▭	▭	▭	▭
Knows and can produce the handshapes of 20 or more letters	▭	▭	▭	▭
Knows and can produce the full fingerspelling alphabet	▭	▭	▭	▭
Can understand simple words when fingerspelled by someone else	▭	▭	▭	▭
Can translate written letters to fingerspelled letters	▭	▭	▭	▭
Can fingerspell a few simple words like "Mommy" or "dog"	▭	▭	▭	▭
Can fingerspell 50 to 100 words	▭	▭	▭	▭
Makes appropriate vocal sounds when producing individual fingerspelled letters	▭	▭	▭	▭
Can fingerspell words when presented with the ASL translations of those words	▭	▭	▭	▭

Can combine fingerspelled words and signs when expressing full sentences	☐	☐	☐	☐

SECTION F SPECIAL EDUCATION AND SERVICES

F1. When your child moved from the program serving children under age 3 to his/her preschool program, how much did you understand the procedures related to this transition?

☐ Not at all ☐ A little ☐ Fairly well ☐ A great deal

F2. When your child moved into the preschool program, would you say he/she received more services, less services, or about the same amount of services that he/she received in the program for children under age 3?

- ☐ More
- ☐ Less
- ☐ Same
- ☐ Don't know

F3. Who first referred your child for preschool special education services?

- ☐ Early intervention program
- ☐ Child find
- ☐ Parent
- ☐ Physician
- ☐ Head start
- ☐ Preschool staff
- ☐ Health department
- ☐ Other family member/friend
- ☐ Child care program
- ☐ Other (specify:______________________________)
- ☐ Don't know

F4. Indicate the age at which your child first received preschool education services.

- ☐ 1 year of age
- ☐ 2 years of age
- ☐ 3 years of age
- ☐ 4 years of age
- ☐ 5 years of age
- ☐ N/A (child did not have preschool education services)

F5. How much effort did it take to find out how to get preschool special education services started through the school system (e.g., asking people about what could be done for your child, asking about testing, calling places to try to get information about services)?

☐ A lot of effort ☐ Some effort ☐ Little effort ☐ No effort at all ☐ Don't know/ no opinion

F6. About how old was your child when your family tried to get preschool special education services for him/her?

Months _____ Years ________

F7. Did your child have an Individual Family Service Plan (*IFSP: a legal document or written plan developed to determine what services a young child with disabilities needs*) for the service(s) he/she received before the age of three?

☐ Yes ☐ No ☐ Don't know

F8. Within the past two months, did your child have an Individualized Education Plan (*IEP: establishes goals for your child and identifies special education services to help your child meet those goals*) or did he/she receive special education or other services for a special need or disability, such as speech therapy, physical therapy, or some Other help?

- Yes
- No
- Don't know

F9. Does your child currently have a 504 plan (*a documented program of instructional and/or assessment provisions to assist students with special needs who are in a regular education setting, as required by Section 504 of the Vocational Rehabilitation Act*) for classroom accommodations because of his/her special needs?

- Yes
- No
- Don't know

F10. Which of the following statements best describes where your child spends his/her time at school?

- My child spends the entire time in the general education class working only with the general education teaching staff.
- My child spends the entire time in the general education class and specialists come in and work with him/her there.
- My child spends most of the time in the general education class but is taken out of the classroom to receive some special services.
- My child spends some time in the general education class and some time in a special education class for children with special needs.
- My child spends the entire day in a special class for children with special needs.
- Other (specify:____)
- Don't know

F11. Mark all of the following services your child receives in support of instruction:

- My child does not receive services in support of instruction

- Oral transliteration services
- Cued language transliteration services
- Signed transliteration services
- Deaf-blind interpreting services
- Oral/Aural (AVT) services
- Vision services/O & M services
- Counseling services, including rehabilitation counseling

(F11 con't)

- Sign language instruction
- CART, C-print, Typewell
- Itinerant teacher services
- Classroom paraprofessional services
- Audiology services
- CI mapping
- Tutoring services
- Adaptive PE
- Speech-language pathology
- Occupational/physical therapy
- School nurse/medical services
- Social work services
- Psychological services
- Recreation, including therapeutic recreation
- Transportation
- Resource
- Remedial ASL services
- Other: ________________________

F12. Does a specialist meet with your child's teacher or child care provider to show the teacher how to work with your child?

- Yes
- No
- Don't know

F13. Does a specialist come to the program and take your child out of class to provide special services?

- Yes
- No
- Don't know

F14. Does a specialist come to your home to work with your child or a family member?

- Yes
- No

F15. Is there any other way that your child receives services? ▭ No ▭ Yes (specify: ____________________)

F16. Are there any special education services or therapies that you think your child should be getting through the program or the school system, but isn't?

▭ Yes ▭ No ▭ Don't know

a. If yes, what therapy or services do you think your child needs, but isn't getting?

Specify:____

F17. How would you rate the general quality of the special education and therapy services your child is getting through the program or school system?

▭ Excellent ▭ Good ▭ Fair ▭ Poor ▭ Some are okay; some are not okay

F18. During the last year, did you or another adult in your child's household go to a meeting about an IEP (*Individualized Education Plan*) or IFSP (*Individual Family Service Plan*) for your child?

▭ Yes ▭ No

F19. How do you feel about your family's involvement in the decisions about your child's IEP/IFSP?

▭ We wanted to be involved more
▭ We were involved about the right amount
▭ We wanted to be involved less
▭ We have no opinion

F20. For each of the statements below, indicate whether you strongly agree, agree, disagree, or strongly disagree with the statement.

	Strongly Agree	Agree	Disagree	Strongly Disagree	No Opinion
I have good feelings about the professionals who work with my child and family.	▭	▭	▭	▭	▭
Professionals who work with my child respect the values and cultural background of my family.	▭	▭	▭	▭	▭
Professionals who work with my child ignore my opinions.	▭	▭	▭	▭	▭
Professionals who work with my child make me feel optimistic and hopeful about my child's future.	▭	▭	▭	▭	▭
Professionals who work with my child respect my preferred mode/style of communication.	▭	▭	▭	▭	▭

SECTION G SCHOOL ADJUSTMENT

G1. Thinking about the program(s) your child is currently in,

	Very well	Pretty well	Not very well	Not well at all	Don't know
a. How well you would say he/she gets along with other children at his/her program(s)?	▭	▭	▭	▭	▭
b. How well you would say he/she gets along with teachers at his/her program(s)?	▭	▭	▭	▭	▭

G2. Has your child had any of the following things happen to him/her at his/her program(s)?

	Yes	No	Don't know
a. Has he/she been bullied or picked on by other children or made to do things like give someone money, either at school or on the way to or			

	from school??	__	__	__
b.	Has he/she been physically attacked or involved in fights?	__	__	__
c.	Has he/she been teased or called names?	__	__	__
d.	Has he/she had things stolen from his/her desk, or other places at school?	__	__	__

G3. Thinking about the current school year, rate your satisfaction with the following:

	Very Dissatisfied		Undecided		Very Satisfied
a. The program(s) your child attends	1	2	3	4	5
b. His/her teacher	1	2	3	4	5
c. The services he/she has received	1	2	3	4	5
d. How well you are kept informed about your child's behavior and progress	1	2	3	4	5

G4. Does your child spend time with deaf children in his/her program(s)? __ Yes __ No __ Don't know

G5. Does your child spend time with children in his/her program(s) who are not deaf? __ Yes __ No __ Don't know

G6. During the current school year, have you or another adult in the household done any of the following at your child's program or school?

a.	Attended a general school meeting such as back to school night, or a meeting of a parent-teacher organization?	__ Yes	__ No
b.	Attended a school or class event, such as a play, sports event, or science fair?	__ Yes	__ No
c.	Volunteered in your child's classroom for at least 30 minutes?	__ Yes	__ No
d.	Helped with field trips or other special events?	__ Yes	__ No

(G6 con't)

e.	Attended parent-teacher conferences?	__ Yes	__ No
f.	Participated in fundraising activities?	__ Yes	__ No
g.	Communicated regularly with parents of children in your child's class, either in person or on the phone or with a communication book?	__ Yes	__ No
h.	Met your child's teacher?	__ Yes	__ No

G7. During the current school year, has your child's teacher or someone else from your child's school or program done any of the following?

a.	Sent your family personal notes?	__ Yes	__ No
b.	Sent newsletters, memos, or notices addressed to all parents?	__ Yes	__ No
c.	Called you on the phone?	__ Yes	__ No
d.	Sent e-notes from school?	__ Yes	__ No

G8. Does your child attend the neighborhood school or the same school as the other children in the neighborhood?

__ Yes __ No

G9. Think about your child's experience at his/her school or program during the current school year and rate the following statements:

	Strongly Agree	Agree	Disagree	Strongly Disagree	No Opinion
My child enjoys school	__	__	__	__	__
My child's teachers maintain good discipline in the classroom	__	__	__	__	__
Most students and teachers respect each other	__	__	__	__	__

The principal and assistant principal maintain good discipline at my child's school	▭	▭	▭	▭	▭
The school is good at meeting my child's individual needs	▭	▭	▭	▭	▭

G10. How far in school do you expect your child to go?

I expect my child to…

- ▭ attend some high school
- ▭ graduate from high school
- ▭ attend some college or take post secondary vocational courses
- ▭ receive a 2- or 3-year college degree (AA DEGREE) or vocational school diploma
- ▭ earn a 4-year college degree (BA, BS DEGREE)
- ▭ earn a graduate degree (MA, MBA, Ph.D., JD, MD)

The following section includes a wide variety of beliefs that people have regarding deaf children and various educational/ communication philosophies. Some of these beliefs or statements you may agree with—maybe even strongly agree with. Others of these beliefs or statements you may disagree with; some you may think are completely wrong. We are interested in understanding what people think about different education and communication philosophies. We appreciate your honest opinions in this section. There are no right or wrong answers—only your opinions.

SECTION H BELIEFS ABOUT DEAF EDUCATION

Indicate your level of disagreement or agreement with each statement.

	Strongly Disagree	Disagree	No Opinion	Agree	Strongly Agree
1. Children less than six years old should receive general services for developmental delays and not be labeled with a specific disability.	1	2	3	4	5
2. Learning in the regular classroom through an interpreter produces higher levels of learning than in a deaf education classroom with a teacher of the deaf.	1	2	3	4	5
3. Learning two languages would be too great a challenge for young deaf or hard of hearing children.	1	2	3	4	5
4. Deaf or hard of hearing children should enter hearing classrooms as soon as possible in order for them to learn grade-level information along with their hearing peers.	1	2	3	4	5
5. Talking and signing at the same time provides children access to both a visual and an auditory language.	1	2	3	4	5
6. Young deaf and hard of hearing children can learn fingerspelling as infants.	1	2	3	4	5
7. Deaf or hard of hearing infants and toddlers should receive early intervention services primarily through training provided to their parents/guardians in natural environments.	1	2	3	4	5
8. Many hearing parents do not learn to sign because they have chosen another communication approach for their child.	1	2	3	4	5
9. It is a positive experience to have parents of deaf or hard of hearing children meet deaf adults.	1	2	3	4	5
10. Talking combined with Cued Speech (CS) and/or speechreading provides children visual and auditory access to language.	1	2	3	4	5
11. Because sign language hinders the development of listening and talking, young deaf or hard of hearing children should be allowed to develop spoken language initially *without* the influence of signs	1	2	3	4	5
12. If children have early access to spoken language through hearing without visual supports then they will development better later language skills.	1	2	3	4	5
13. If children use hearing aids, they will learn language through their residual hearing regardless of the level of hearing loss.	1	2	3	4	5
14. Using any visual supports while talking is confusing and hinders the development of auditory access to language.	1	2	3	4	5

15. ASL is a visual language and people should not use ASL and speak at the same time.	1	2	3	4	5
16. With amplification (hearing aids or a cochlear implant) and focused early intervention, deaf children will be able to attend regular classes in elementary school without needing an interpreter or transliterator.	1	2	3	4	5
17. New technologies (e.g., cochlear implants) are effective in producing normal-like hearing ability in deaf children.	1	2	3	4	5
18. Language can be learned visually; therefore, American Sign Language (ASL) is an appropriate communication approach for young children.	1	2	3	4	5
19. Cued Speech is an appropriate communication approach for young children.	1	2	3	4	5
20. Families must focus on a child's medical diagnosis and concentrate on therapeutic interventions during the first three years.	1	2	3	4	5
21. Hearing parents cannot learn ASL; therefore, it is much more effective to help them learn English-based signs or Cued Speech.	1	2	3	4	5
22. Hearing parents cannot learn ASL; therefore, the focus should be on the child's oral language skills.	1	2	3	4	5
23. Parents must make choices about which communication approach to use with their young child.	1	2	3	4	5

	Strongly Disagree	Disagree	No Opinion	Agree	Strongly Agree
24. Efforts initially should focus on medical interventions in order to try to reduce the negative effects of hearing loss.	1	2	3	4	5
25. If children have early access to spoken language through residual hearing and/or vision (e.g., speechreading, Cued Speech) they will develop better later language skills.	1	2	3	4	5
26. If children have early access to sign, they will develop better later language skills.	1	2	3	4	5
27. Many hearing parents do not learn to sign because they are overwhelmed by other demands on their resources (e.g., other children, finances, other conditions of the child).	1	2	3	4	5
28. Cued Speech, because it provides an accurate visual representation of oral language, can map the brain of young deaf and hard of hearing children, thus giving them an advantage for later developing literacy.	1	2	3	4	5
29. All deaf or hard of hearing children should be educated in the regular classroom with hearing peers regardless of age.	1	2	3	4	5
30. ASL, because it is a visual language, can map the brain of young deaf and hard of hearing children thus giving them an advantage for later developing literacy.	1	2	3	4	5
31. Enrollment in a residential school for the deaf should occur as early as possible.	1	2	3	4	5
32. Academic content can be best learned through ASL.	1	2	3	4	5
33. Academic content can be best learned through the language in which the child will be reading so that they will have access to the same vocabulary and language skills in print and in class.	1	2	3	4	5
34. Parents and teachers of deaf or hard of hearing children should use a combination of all techniques in order to make sure the child is not limited by one approach.	1	2	3	4	5
35. Enrollment in a residential school for the deaf should only be selected when all other placements have failed (e.g., hearing preschool, regular kindergarten, public school mainstreamed classrooms)	1	2	3	4	5
36. Deaf and hard of hearing children can learn ASL.	1	2	3	4	5
37. Deaf and hard of hearing children can learn English if made accessible through a combination of residual hearing, speechreading and Cued Speech as infants.	1	2	3	4	5

38. Being able to read and write is more important than being able to listen and speak.	1	2	3	4	5
39. Learning American Sign Language isolates young children from the hearing world.	1	2	3	4	5
40. Being a member of a Deaf community with a unique culture and language enriches one's life.	1	2	3	4	5
41. A bilingual environment that includes ASL provides full access to language and communication.	1	2	3	4	5
42. Deaf and hard of hearing children's behavior problems come mostly from frustration caused by lack of communication.	1	2	3	4	5
43. Deaf and hard of hearing children can become fluent in English (reading and writing) if given early access to language through ASL in the first year of life.	1	2	3	4	5
44. Special schools for the deaf provide a language-rich educational experience that cannot be replicated in a public school.	1	2	3	4	5
45. Deaf and hard of hearing children who do not have access to ASL when young struggle academically throughout their lives.	1	2	3	4	5
46. Deaf and hard of hearing children can become fluent in English (reading and writing) if given access to spoken language through hearing, speechreading, and/or Cued Speech in the first year of life.	1	2	3	4	5
47. Full access to language and communication is possible for deaf children without the use of ASL.	1	2	3	4	5

SECTION I FAMILY/HOUSEHOLD DEMOGRAPHICS

I1 Which of the following best describes your **current employment situation**?

- ☐ Working full time (35 hours/week or more
- ☐ Working part-time
- ☐ Primarily a student
- ☐ Not working, but actively looking for employment
- ☐ Not working, and not actively looking for employment
- ☐ Retired
- ☐ Other: (specify: ________________________________)

I2 What is the highest level of education completed by the following people?

	Mother	Father	Other (specify: ____________)
a. less than high school, with no GED	☐	☐	☐
b. high school diploma or GED	☐	☐	☐
c. some college/post secondary vocational courses	☐	☐	☐
d. 2- or 3-year college degree (AA degree) or vocational school diploma	☐	☐	☐
e. 4-year college degree (BA, BS degree)	☐	☐	☐
f. some graduate work/no graduate degree	☐	☐	☐
g. graduate degree (MA, MBA, Ph.D., JD, MD)	☐	☐	☐

I3 Do you, or does anyone in your household, currently receive money for your child from the Supplemental Security Income (SSI) program? ☐ Yes ☐ No

I4 Is your child eligible to participate in the free or reduced lunch program at school? ☐ Yes ☐ No

I5 Are any children in your household eligible to participate in the free or reduced lunch program at school?

☐ Yes ☐ No

I6. Do you own your home? ☐ Yes ☐ No

I7. Do you rent your home? ☐ Yes ☐ No

I8. How many rooms in your home? ____________

I9. Household income in the past year:

- ☐ Less than $15,000
- ☐ $15,001 to $25,000
- ☐ $25,001 to $30,000
- ☐ $30,001 to $35,000
- ☐ $35,001 to $40,000
- ☐ $40,001 to $45,000
- ☐ $45,001 to $50,000
- ☐ More than $50,000

Because this is a longitudinal study, we want to be able to contact you again next year to see how you and your child are doing. We want to make sure we don't lose track of you. Please give us permanent contact information by which we can always contact you.

Your e-mail address [please print]: ____

Your cell phone number: ____

Name, address, and phone number of someone who does not currently live with you, but who is likely to know where you are if you move?

First Name ______________ Last Name ______________

What is this person's relationship to this child? ______________

Street Address ______________ Apt # ______________

City ______________ State ______________ Zip ______________

Phone: ( ) ______________ Email: ______________

If you are a foster parent to this child, Is there someone else who would know where your child has moved if she/he is no longer in your foster care? (Such as the social worker assigned to your child.)

First Name ______________ Last Name ______________

Street Address ______________ Apt # ______________

City ______________ State ______________ Zip ______________

Phone: ( ) ______________ Email: ______________

Is there someone else who would know where you are if you move?

First Name ______________ Last Name ______________

Street Address ______________ Apt # ______________

City ______________ State ______________ Zip ______________

Phone: ( ) ______________ Email: ______________

Thank you very much for taking time to answer these questions and helping us with this important study.

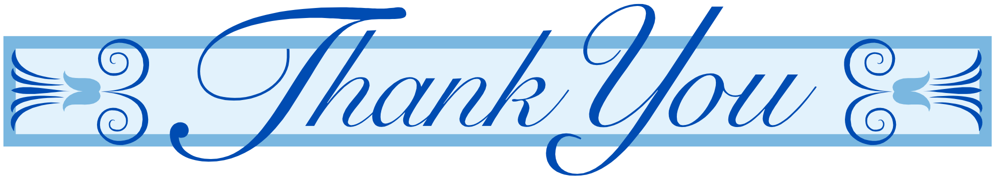

Supplement: S1 Survey — (PDF) [file pone.0229591.s002.pdf]
